# PARP inhibition-associated heterochromatin confers increased DNA replication stress and vulnerability to ATR inhibition in SMARCA4-deficient cells

**DOI:** 10.1038/s41420-025-02306-1

**Published:** 2025-01-28

**Authors:** Kimiyoshi Yano, Megumi Kato, Syoju Endo, Taichi Igarashi, Ryoga Wada, Takashi Kohno, Astrid Zimmermann, Heike Dahmen, Frank T. Zenke, Bunsyo Shiotani

**Affiliations:** 1 Laboratory of Genome Stress Signaling, National Cancer Center Research Institute, Chuo-ku, Tokyo 104-0045 Japan; 2 Department of Cellular and Molecular Biology, Graduate School of Biomedical and Health Sciences, Hiroshima University, Minami-ku, Hiroshima-city, Hiroshima 734-8553 Japan; 3 Department of NCC Cancer Science, Health Sciences and Biomedical Engineering, Graduate School of Medical and Dental Sciences, Tokyo Medical and Dental University, Bunkyo-ku, Tokyo 113-8510 Japan; 4 Department of NCC Cancer Science, Division of Integrative Molecular Biomedicine, Biomedical Sciences and Engineering, Graduate School of Medical and Dental Sciences, Tokyo Medical and Dental University, Bunkyo-ku, Tokyo 113-8510 Japan; 5 Department of Biosciences, School of Science, Kitasato University, Minami-ku, Sagamihara-city, Kanagawa 252-0373 Japan; 6 Department of Pharmacology, Hoshi University School of Pharmacy and Pharmaceutical Sciences, Shinagawa-ku, Tokyo 142-8501 Japan; 7 Division of Genome Biology, National Cancer Center Research Institute, Chuo-ku, Tokyo 104-0045 Japan; 8 Research Unit Oncology, The healthcare business of Merck KGaA, Frankfurter Str. 250, 64293 Darmstadt, Germany; 9 Department of Genome Stress Signaling, Institute of Medical Science, Tokyo Medical University, Shinjuku-ku, Tokyo 160-0023 Japan

**Keywords:** Targeted therapies, Stalled forks

## Abstract

DNA replication stress (RS), a prevalent feature of various malignancies, arises from both genetic mutations and genotoxic exposure. Elevated RS levels increase the vulnerability of cancer cells to ataxia telangiectasia and Rad3-related kinase inhibitors (ATRis). Here, we screened for DNA damage response inhibitors that enhance ATRi-induced cytotoxicity using SWI/SNF complex-deficient cells and identified a potent synergy between ATRi and poly(ADP-ribose) polymerase inhibitor (PARPi), particularly in SMARCA4-deficient cells. PARP inhibition triggers chromatin changes, namely elevated histone H3 at lysine 9 di-methylation (H3K9me2), a hallmark of facultative heterochromatin, increasing dependence on ATR activity for replication fork progression and cell survival. Interestingly, SMARCA4 deficient cells, intrinsically vulnerable to replication stress, exhibited exacerbated DNA damage upon combined ATRi and PARPi treatment in a Mre11- and Mus81-mediated manner. In vivo, combined treatment with intermittent ATRi and continuous PARPi showed greater inhibition of tumor growth than ATRi alone in SMARCA4-deficient lung adenocarcinoma xenograft models. These findings demonstrate that PARPi-induced heterochromatin amplifies RS and ATRi susceptibility, providing a potential rationale for therapeutic strategies targeting SMARCA4-deficient tumors.

## Introduction

Ataxia telangiectasia and Rad3-related (ATR) kinase, which is a master regulator in response to DNA replication stress (RS), regulates cell cycle checkpoints and facilitates DNA repair via its substrates [1–3]. RS is defined as the slowing or stalling of DNA replication fork progression, and persistent RS leads to genomic instability and lethality if unrepaired. Many cancer cells harbor genetic mutations that generate various obstacles to replication fork progression (e.g., heterochromatin or transcribed genes) or compromise the DNA damage response (DDR), leading to incomplete DNA repair, thereby disrupting normal DNA replication and increasing the levels of RS [4–6]. Importantly, when fork progression is interrupted, the ATR signaling pathway promotes the sustainability of fork progression, which is termed RS tolerance, by protecting and restarting stalled forks [7–14]. ATR also activates the G2/M cell cycle checkpoint through activation of Chk1 kinase, which provides time to complete DNA replication and repair. Following ATR inhibition, RS deteriorates due to increased fork instability and widespread activation of dormant replication origins, leading to irreversible collapse of DNA replication, known as replication catastrophe [15]. In addition, ATR inhibition forces cells to prematurely enter mitosis with under-replicated and/or unrepaired DNA, triggering mitotic catastrophe [16]. These observations suggest that ATR-dependent RS responses contribute to cancer cell survival and therapeutic resistance, and that ATR is a promising target for cancer therapy. Accumulating evidence has shown that ATR inhibition is selectively toxic to cancer cells harboring high levels of RS [3, 17, 18]. Thus, clinical trials are currently being conducted to evaluate the efficacy of ATR inhibitors (ATRis) as monotherapy or in combination with other agents based on biomarkers, if available. However, effective therapeutic approaches using ATRis have not yet been established for its successful clinical development.

Switch/sucrose-nonfermentable (SWI/SNF) chromatin remodeling complexes regulate gene transcription and DDR by controlling nucleosome positioning and are associated with human cancer [19, 20]. More than 20% of human cancers harbor a mutation in one of the subunits of the SWI/SNF complex, many of which are loss-of-function mutations. Evaluating the preclinical efficacy of ATRis by us and others identified deficiency in SMARCA4, a core component of the SWI/SNF complex, as a predictive biomarker of ATRi efficacy [21, 22]. Transient depletion of SMARCA4 increases R-loop-mediated transcription-replication conflicts [23]. Additionally, constitutive loss of SMARCA4 in lung adenocarcinoma (LUAD) cells leads to heterochromatin formation [21]. Both contribute to problems in fork progression and render these cells dependent on ATR activity. Defects in other subunits of SWI/SNF complexes, including ARID1A and PBRM1, have also been reported to induce RS through distinct mechanisms, which increase reliance on ATR checkpoint activity [24, 25]. Genome-wide CRISPR or si/shRNA screens to discover comprehensive ATRi biomarkers have identified several candidates for genetic biomarkers such as RNASEH2 and ERCC1, but they share an only limited gene list, and SWI/SNF components are not among the top candidates except for ARID1A in one study [26, 27]. This is likely because these screening models assess the response to ATRi under the immediate effects of knockdown or knockout, which occurs over a relatively short period. Therefore, evaluating ATRi in established SWI/SNF-deficient cancer cells that have tolerated chronic impairments, including RS, over a long period of time could provide more clinically relevant insights.

Recent clinical trials and preclinical studies have increasingly evaluated synergies with inhibitors of DDR factors as partner drugs to enhance the efficacy of ATRi. One of the most promising is an inhibitor of poly(ADP-ribose) polymerase (PARP), which facilitates the repair of single-stranded DNA breaks (SSBs) [28]. PARPis create S-phase DNA damage by PARP trapping, as well as by preventing repair of SSBs, double-strand breaks (DSBs), and stalled forks [29]. ATRis prevent cellular recovery from PARPi-induced DNA damage and cause rapid cell death by premature mitotic entry with unrepaired DNA damage in BRCA1/2- and ATM-mutant cancer cells [30, 31]. Moreover, recent studies have reported that upon PARP inhibition, ATRi preferentially exacerbates nascent DNA degradation from single-stranded DNA (ssDNA) gaps via PrimPol-mediated repriming in BRCA1/2-deficient cells [32]. Although these studies indicate that PARPis synergize with ATRis by abrogating DNA repair, it is not yet well understood whether PARPis increase the sources of RS and how those obstacles affect fork progression in the presence of ATRis. In addition, inhibitors of ataxia telangiectasis mutated (ATM) and DNA-dependent protein kinase (DNA-PK), which respond to DSBs and promote DNA repair and cell cycle checkpoints [33], are also potential combination partners. *ATM* is frequently mutated in cancer, and ATM-deficient cancer cells are more sensitive to ATRis [34–38], probably because of the interdependence between ATM and ATR in response to secondary DSBs induced by RS. Alternatively, combined ATRi and DNA-PKi radiosensitizes tumor cells independently of the p53 status [39]. These findings highlight that ATRis combined with DDRis could be an effective therapeutic approach.

To explore the synthetic lethal relationship between ATR inhibition and loss of function of the SWI/SNF complex, we evaluated SMARCA4-, ARID1A-, and PBRM1-deficient LUAD cells using a novel potent and selective ATRi (tuvusertib) and further characterized the synergistic effects of ATRi in combination with ATMi (lartesertib), DNA-PKi (peposertib), and PARPi (niraparib). Our results showed that histone H3 at lysine 9 dimethylation (H3K9me2)-associated heterochromatin induced by PARPi increased the dependence on ATR activity for fork progression and cell survival, resulting in a greater susceptibility to ATRi in SMARCA4-deficient cells in vitro and in xenograft models. In summary, our findings provide useful insights into the mechanism of action of the ATRi/PARPi combination and an attractive approach for potential ATRi therapy for SMARCA4-deficient LUAD.

## Results

### ATRi/PARPi combination exhibits greater synergy and sensitivity particularly in SMARCA4-deficient LUAD cells

**Fig. 1:**
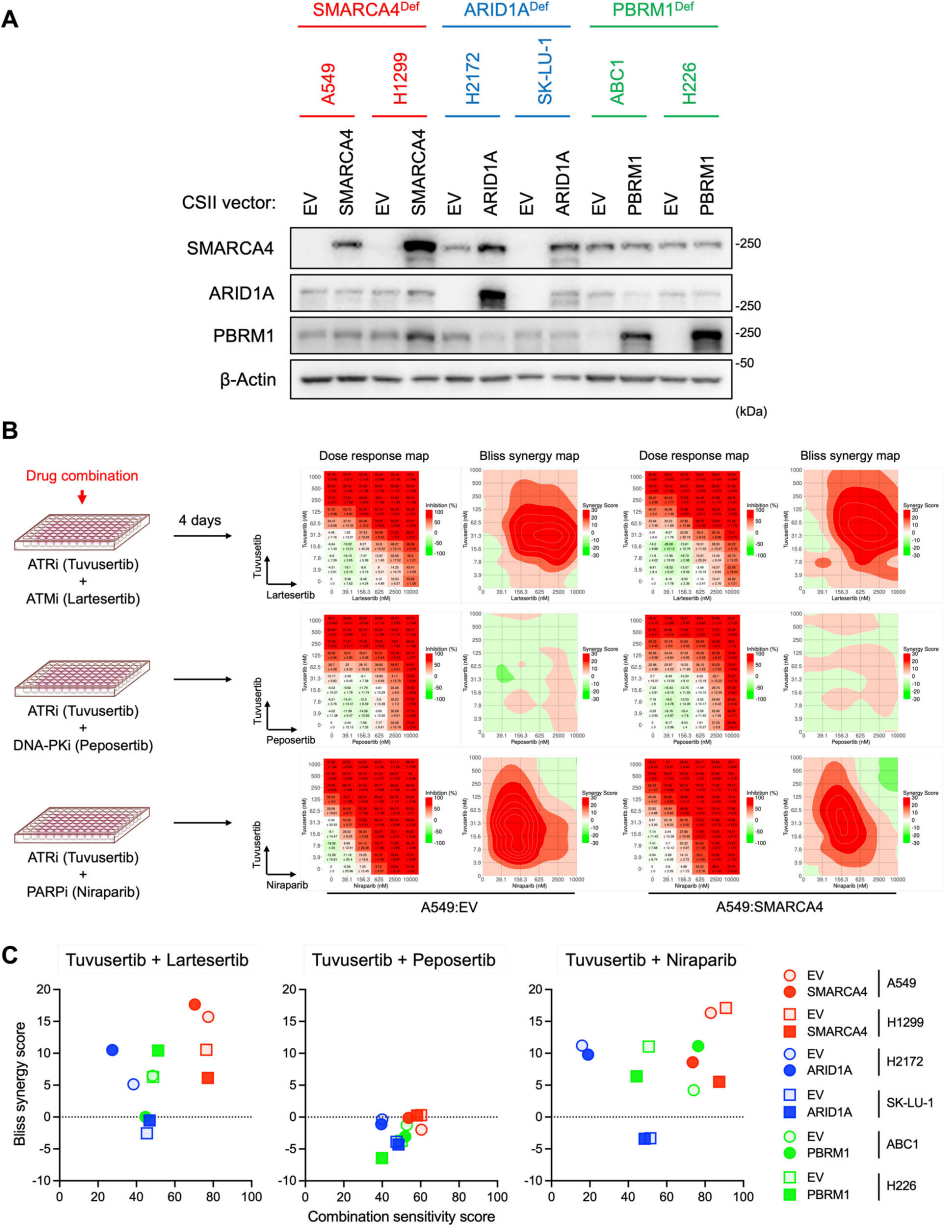
Cell viability screening for ATRi in combination with ATMi, DNA-PKi, or PARPi in SWI/SNF-deficient LUAD cells. **A** LUAD cell lines harboring deficiency of SMARCA4 (A549 and H1299), ARID1A (H2172 and SK-LU-1), and PBRM1 (ABC1 and H226) were constitutively expressed the corresponding wild-type proteins via lentiviral cDNA expression system. Expression of SMARCA4, ARID1A, and PBRM1 proteins was analyzed by western blotting. β-Actin was detected as loading control. **B** Cell viability screening for tuvusertib (0, 3.9–1000 nM) in combination with lartesertib (0, 39.1–10000 nM), peposertib (0, 39.1–10000 nM), or niraparib (0, 39.1-10000 nM). Cells were treated with these combinations for 4 days, then a cell viability assay was performed. Using SynergyFinder plus, dose response and Bliss synergy maps in A549 cells are shown in Fig. 1B, and other cells are shown in Fig. S1E–G. These maps indicate the mean of three independent experiments. **C** Combination sensitivity score-Bliss synergy score plots of tuvusertib in combination with lartesertib, peposertib, or niraparib in the cell viability screening shown in Fig. 1B.

Given that deficiencies of SWI/SNF components, including SMARCA4, ARID1A, and PBRM1, are biomarker candidates for ATRi efficacy [21, 22, 24, 25], we hypothesized that ATRi combo-therapy would be more effective than monotherapy in SWI/SNF-deficient LUAD cells. We performed cell viability screening to assess the synergistic cytotoxicity of ATRi in combination with DDRis in LUAD cell lines harboring deficiencies in SMARCA4 (A549 and H1299), ARID1A (H2172 and SK-LU-1), and PBRM1 (ABC1 and H226) (Table S1). These SWI/SNF-deficient LUAD cells were complemented with the corresponding wild-type (WT) proteins via a lentiviral cDNA expression system (Fig. 1A), allowing us to analyze the effect of the presence of these proteins on various treatments in the same genetic background. In this assay, we assessed a potent, selective, small-molecule inhibitor of ATR (tuvusertib), which inhibited ATR autophosphorylation at Ser1989 and Chk1 phosphorylation at Ser345 upon camptothecin (CPT)-induced DNA damage (Fig. S1A). ATMi (lartesertib), DNA-PKi (peposertib), and PARPi (niraparib) inhibited CPT-induced Chk2 phosphorylation at Thr68, RPA32 phosphorylation at Ser4/8, and PARylation, respectively, confirming their mechanisms of action (Fig. S1B–D). We treated cells with these inhibitors in combinations and analyzed cell viability data using SynergyFinder Plus [40], which displayed dose-response and synergy maps (Figs. 1B, S1E–G) and summarized the combination sensitivity score-Bliss synergy score plots for each combination (Fig. 1C). Lartesertib and niraparib, but not peposertib, showed synergistic effects in combination with tuvusertib in most of the cancer cell lines tested (Fig. 1C). Notably, the tuvusertib/niraparib combination showed higher synergy scores in SMARCA4-deficient cells (Bliss synergy score; A549:EV = 16.33 and H1299:EV = 17.1) than in ARID1A- and PBRM1-deficient cells, accompanied by the highest sensitivity scores (Combination sensitivity score; A549:EV = 82.98 and H1299:EV = 90.81) (Fig. 1C). Consistently, tuvusertib alone showed greater sensitivity in SMARCA4-deficient cells than in other cells (Fig. S1H). These results suggested that the functional status of *SMARCA4* is associated with tuvusertib- and tuvusertib/niraparib-induced cytotoxicity, in contrast to *ARID1A* and *PBRM1* defects. Importantly, complementary expression of SMARCA4 in A549 and H1299 cells lowered both scores, confirming that SMARCA4 deficiency enhanced tuvusertib/niraparib-induced cytotoxicity (Fig. 1C). Although lartesertib showed promising combination sensitivity and Bliss synergy scores in SMARCA4 deficient cells, they were independent of SMARCA4 expression (Fig. 1C). Taken together, these findings indicate potent synergy between ATRi and PARPi, particularly in SMARCA4-deficient LUAD cells.

### Cytotoxicity and DNA damage sensitization caused by ATRi/PARPi combination in SMARCA4 knockout cells

**Fig. 2:**
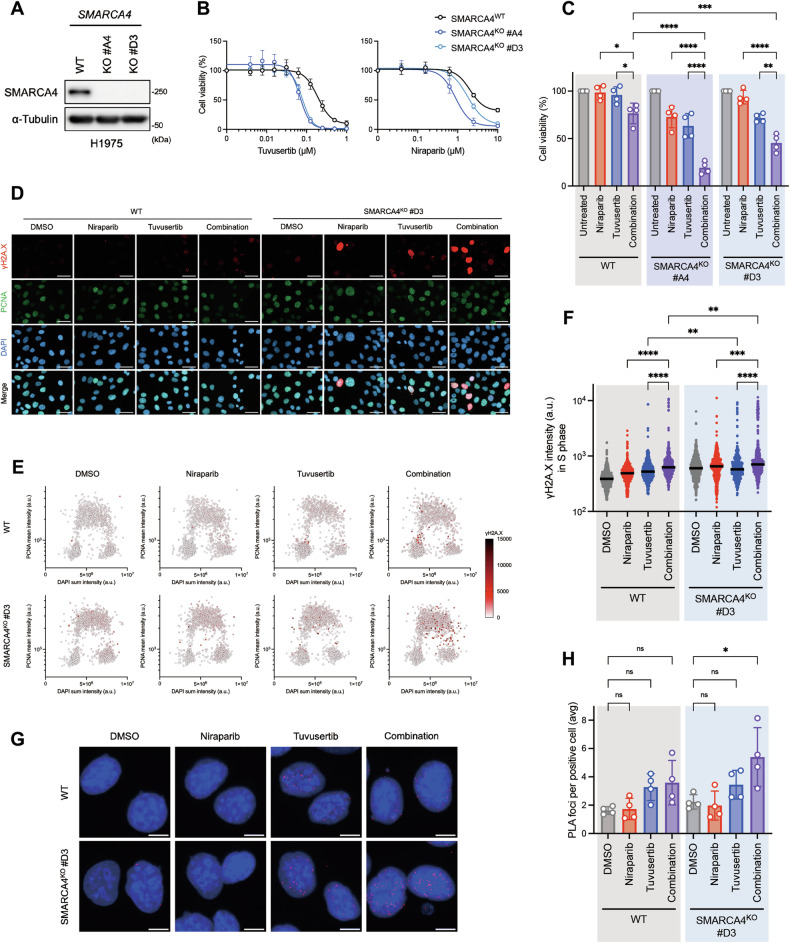
SMARCA4 deficiency sensitizes DNA damage and cytotoxicity upon ATRi/PARPi combination. **A** Western blot analysis of SMARCA4 wild-type (WT) and knockout (KO) H1975 cells. α-Tubulin was detected as loading control. **B** Cell viability for tuvusertib or niraparib alone. Cells were treated with tuvusertib (0, 3.9–1000 nM) and niraparib (0, 39–10000 nM) combination for 6 days, then a cell viability assay was performed. These graphs indicate the mean ± SD of four independent experiments. **C** Cell viability at 62.5 nM tuvusertib and 625 nM niraparib in the experiment shown in Fig. 2B. The graph indicates the mean ± SD of four independent experiments and statistical significance by one-way ANOVA test with Tukey’s multiple comparisons test. *P*-value: * ≤ 0.05, ** ≤ 0.01, *** ≤ 0.001, and **** ≤ 0.0001. **D** Representative images of each samples show each and merged images of DAPI (blue), PCNA (green), and γH2A.X (red). Cells were treated with 62.5 nM tuvusertib and 625 nM niraparib each alone and combination for 48 h, then carried on immunofluorescence analysis. Scale bars indicate 50 µm. **E** QIBC plots of DAPI sum intensity versus PCNA mean intensity, with γH2A.X mean intensity (color scale) per nuclei in the experiment shown in Fig. 2D. These plots indicate 2000 cells. **F** Dot plot of γH2A.X mean intensity per nuclei in S phase cells in the experiment shown in Fig. 2D. PCNA-positive cells were defined as S phase cells. The graph indicates the median of 500 cells and statistical significance by Kruskal–Wallis test with Dunn’s multiple comparisons test. *P*-value: ** ≤ 0.01, *** ≤ 0.001, and **** ≤ 0.0001. **G** Representative images of each samples show merged images of DAPI (blue) and PLA foci of EdU and γH2A.X (red). Cells were treated with 62.5 nM tuvusertib and 625 nM niraparib each alone and combination for 48 h, then carried on PLA analysis. Scale bars indicate 10 µm. **H** Quantification of the average number of PLA foci per positive nuclei (foci ≥ 1) in S phase cells. The graph indicates the mean ± SD of four independent experiments and statistical significance by one-way ANOVA test with Tukey’s multiple comparisons test. *P*-value: not significant (ns) > 0.05 and * ≤ 0.05.

To validate the screening results, two clones of SMARCA4 knockout (SMARCA4^KO^) cells derived from H1975 cells (SWI/SNF^WT^ LUAD cell line) were established to assess the cytotoxicity of the ATRi/PARPi combination (Fig. 2A). Consistent with SMARCA4-deficient LUAD cells, SMARCA4^KO^ cells were more sensitive to tuvusertib or niraparib alone compared with SMARCA4^WT^ cells (Fig. 2B). The relatively low concentrations of tuvusertib (62.5 nM) and niraparib (625 nM) [41, 42], which exhibited subtle cytotoxic effects on SMARCA4^WT^ cells, showed clear synergistic effects in combination in SMARCA4^KO^ cells compared to each drug alone, but the synergistic effects in SMARCA4^WT^ cells were minimal (Fig. 2B, C). SMARCA4^KO^ cells exhibited a pronounced synergistic response to the combination treatment, with the maximal effect observed at low concentrations of tuvusertib and niraparib (Fig. S2A, B). In contrast, while SMARCA4^WT^ cells also showed synergistic interactions, higher concentrations of the inhibitors were required to achieve a similar level of synergy. These findings suggest that the loss of SMARCA4 sensitizes cells to the combined effects of ATRi and PARPi. To understand the basis of ATRi toxicity, we analyzed the DNA damage-induced γH2A.X signal. Niraparib significantly increased tuvusertib-induced γH2A.X levels in SMARCA4^WT^ S-phase cells, which were significantly exacerbated in SMARCA4^KO^ cells (Fig. 2D–F). These cells became strongly pan-nuclear-positive for γH2A.X in the S phase (Fig. 2D), and were primarily, but not exclusively, found in mid-to-late S phase cells (Fig. 2E). Furthermore, in the Proximity Ligation Assays (PLA) of γH2A.X and EdU, a significant increase in PLA focus formation was observed upon treatment with the niraparib and tuvusertib combination treatment in SMARCA4^KO^ cells (Fig. 2G, H), confirming that γH2A.X is a consequence of fork breakage, an indicator of replication catastrophe-associated DNA damage. Considering these results, the combination of tuvusertib/niraparib could offer a potentially effective therapeutic approach, particularly in SMARCA4-deficient LUAD cells.

### PARPi-induced heterochromatin-dependent RS is associated with ATR dependence for fork progression and cell survival

**Fig. 3:**
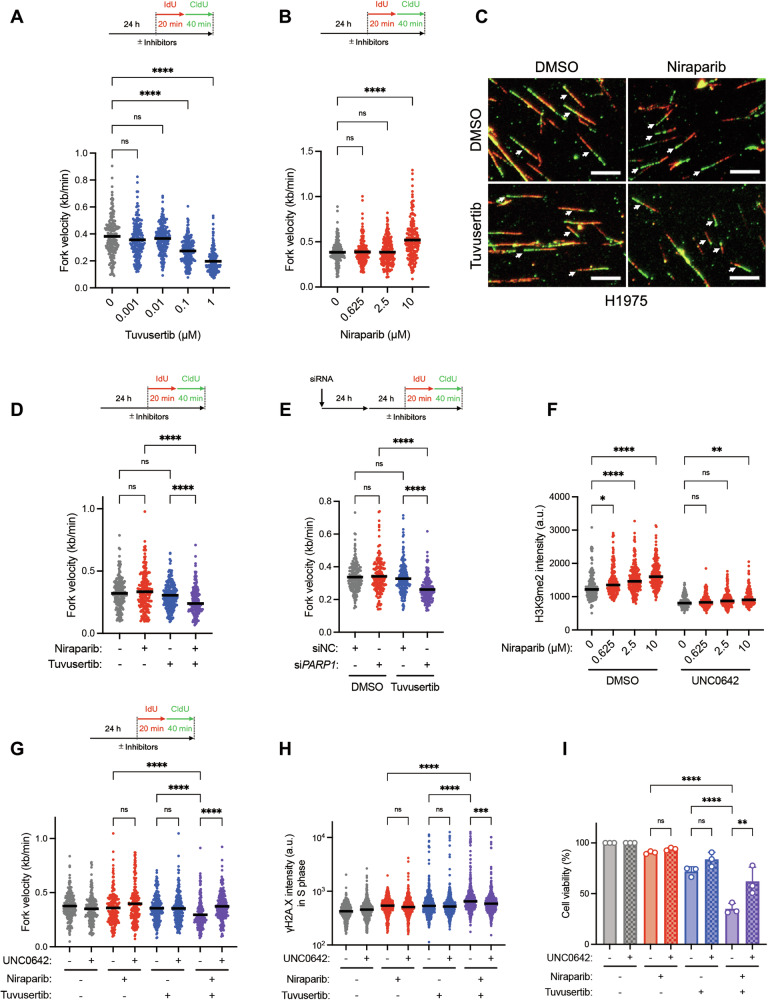
PARP inhibition-induced H3K9me2 accumulation increases ATR dependence for fork progression and cell survival. **A** Dot plot of fork velocity in H1975 cells. Cells were treated with tuvusertib for 24 h prior to IdU and CldU labeling, then were carried on DNA fiber assay. The graph indicates the mean of 200 fibers and statistical significance by Kruskal–Wallis test with Dunn’s multiple comparisons test. *P*-value: not significant (ns) > 0.05 and **** ≤ 0.0001. **B** Dot plot of fork velocity in H1975 cells. Cells were treated with niraparib for 24 h prior to IdU and CldU labeling, then were carried on DNA fiber assay. The graph indicates the mean of 200 fibers and statistical significance by Kruskal–Wallis test with Dunn’s multiple comparisons test. *P*-value: not significant (ns) > 0.05 and **** ≤ 0.0001. **C** Representative immunofluorescent images of DNA fiber assay in H1975 cells. Cells were treated with 10 nM tuvusertib and 2.5 µM niraparib for 24 h prior to IdU (red) and CldU (green) labeling, then a DNA fiber assay was performed. Scale bars indicate 10 µm. **D** Dot plot of fork velocity in the experiment shown in Fig. 3C. The graph indicates the mean of 200 fibers and statistical significance by Kruskal–Wallis test with Dunn’s multiple comparisons test. *P*-value: not significant (ns) > 0.05 and **** ≤ 0.0001. **E** Dot plot of fork velocity in H1975 cells. Cells were transfected with siRNA for 24 h, were treated with 10 nM tuvusertib for 24 h prior to IdU and CldU labeling, then were carried on DNA fiber assay. The graph indicates the mean of 200 fibers and statistical significance by Kruskal–Wallis test with Dunn’s multiple comparisons test. *P*-value: not significant (ns) > 0.05 and **** ≤ 0.0001. **F** Dot plot of H3K9me2 mean intensity per nuclei in H1975 cells. Cells were treated with niraparib and 2 µM UNC0642 for 24 h, then were carried on immunofluorescence analysis. The graph indicates the median of 200 cells and statistical significance by Kruskal–Wallis test with Dunn’s multiple comparisons test. *P*-value: not significant (ns) > 0.05, * ≤ 0.05, ** ≤ 0.01, and **** ≤ 0.0001. **G** Dot plot of fork velocity in H1975 cells. Cells were treated with 10 nM tuvusertib, 2.5 µM niraparib, and 2 µM UNC0642 for 24 h prior to IdU and CldU labeling, then were carried on DNA fiber assay. The graph indicates the mean of 200 fibers and statistical significance by Kruskal–Wallis test with Dunn’s multiple comparisons test. *P*-value: not significant (ns) > 0.05 and **** ≤ 0.0001. **H** Dot plot of γH2A.X mean intensity per nuclei in S phase cells. Cells were treated with 100 nM tuvusertib, 625 nM niraparib, and 2 µM UNC0642 each for 48 h, then an immunofluorescence analysis was performed. PCNA-positive cells were defined as S phase cells. The graph indicates the median of 500 cells and statistical significance by Kruskal–Wallis test with Dunn’s multiple comparisons test. *P*-value: not significant (ns) > 0.05, *** ≤ 0.001, and **** ≤ 0.0001. **I** Cell viability relative to untreated or UNC0642-treated cells in H1975 cells. Cells were treated with 100 nM tuvusertib, 625 nM niraparib, and 2 µM UNC0642 for 6 days, then were carried on cell viability assay. The graph indicates the mean ± SD of three independent experiments and statistical significance by one-way ANOVA test with Tukey’s multiple comparisons test. *P*-value: not significant (ns) > 0.05, ** ≤ 0.01, and **** ≤ 0.0001.

Accumulating evidence has shown that ATRis prevent cellular recovery from PARPi toxicity in BRCA1/2-deficient cancer cells by premature mitotic entry with unrepaired DNA damage and by degrading ssDNA gaps [30, 32], indicating that ATRi/PARPi combination exhibited synergistic cytotoxicity in BRCA1/2-deficient background. However, in the presence of BRCA1/2, the mechanism of synthetic lethality of ATR/PARP inhibition is not well understood. PARPis including niraparib leave trapped PARP1 and unrepaired DNA damage that can become obstacles to fork progression, yet cells treated with PARPis can progress forks under RS. Because ATR plays an important role in RS tolerance by regulating fork protection and/or promoting fork restart, we hypothesized that ATR kinase activity is required for sustained fork progression in PARPi-treated cells, in which ATR and Chk1 were activated in a niraparib concentration-dependent manner (Fig. S3A). To test this hypothesis, we examined the effects of ATR and PARP inhibition on fork progression in SMARCA4^WT^ H1975 cells using a DNA fiber assay. Following drug treatment, we pulse-labeled the cells with 5-Iodo-2’-deoxyuridine (IdU) and 5-Chloro-2’-deoxyuridine (CldU) to monitor the length of the newly synthesized DNA. ATRi causes fork slowdown when used at high concentrations through both direct and indirect effects, including a combination of increased replication stress, fork destabilization, unscheduled origin firing, and the accumulation of DNA damage [1, 7, 15, 43]. Consistently, tuvusertib, at high concentrations (0.1 μM and 1 μM), reduced fork velocity in our experimental conditions (Fig. 3A). In contrast, PARP1 inhibitors have been shown to increase fork velocity in cells treated with 10 μM of the PARPi olaparib [44, 45]. PARP inhibition leads to increased fork speed by bypassing the stalling and repair functions that PARP1 performs at the replication fork, resulting in faster but potentially less stable replication with ssDNA gaps behind forks in a PrimPol-dependent manner [28, 46]. By trapping PARP1 on DNA, PARPi prevents the completion of gap repair until the next S phase, leading to collisions of replication forks with ssDNA gaps and a surge of DSBs in a trans cell cycle manner [47]. Consistent with previous reports, we observed that niraparib also increased fork velocity when used at a high concentration (10 μM) (Fig. 3B). We utilized a relatively low concentration of tuvusertib (10 nM) and niraparib (2.5 µM) which are clinically covered for long period of time [41, 42]. As monotherapy, these had minimal effect on fork velocity and showed that tuvusertib significantly reduced fork velocity in niraparib-treated cells, suggesting that replication forks proceed in an ATR-dependent manner under PARPi-induced RS (Fig. 3C, D). Next, we investigated the source of PARP inhibition-induced RS. To test the possibility whether PARP trapping on chromatin might become an obstacle for the replication fork, we conducted experiments using siRNA against PARP1. Interestingly, tuvusertib had minimal impact on fork velocity in control cells, but significantly reduced fork velocity in PARP1-depleted cells (Figs. 3E, S3B). This suggests that the absence of PARP1 activity, rather than the presence of the PARP1 protein itself, induces replication stress. Furthermore, when niraparib was treated at a high concentration (10 μM), a substantial increase in chromatin-bound PARP1, indicative of PARP1 trapping, was observed. However, this trapping was not apparent at lower concentrations of 625 nM or 2.5 μM (Fig. S3C). Notably, at a low concentration of 100 nM which is even lower than the concentration used in the combination, niraparib almost completely inhibited PARylation induced by PARP1 (Fig. S1D). These findings strongly suggest that the enhanced sensitivity to tuvesertib in the presence of niraparib at bioavailable concentrations primarily due to the inhibition of PARP1 activity, with limited contribution from indirect trans cell cycle effects from ssDNA gaps mediated by PrimPol and PARP1 trapping induced by PARPi.

Heterochromatin, a major contributor to RS, hinders fork progression, thereby increasing ATR dependence for RS tolerance and cell survival [14, 21]. Interestingly, a recent study reported that oncogenic IDH mutations lead to the accumulation of H3K9me2 and an increase in heterochromatin-associated RS, which can be resolved dependently of PARP [48]. Consistent with this report, we observed a concentration-dependent increase in H3K9me2 levels, which was clearly decreased by a potent, selective inhibitor of histone methyltransferase G9a/GLP (UNC0642) (Figs. 3F, S3D), suggesting that PARP is involved in histone modification responsible for chromatin dynamics [49]. Moreover, UNC0642 clearly restored the reduction in fork velocity in tuvusertib/niraparib-treated cells (Fig. 3G). Although many secondary changes may contribute to the rescuing effect of UNC0642 after 24 h of treatment, the primary effect of UNC0642—reducing heterochromatin—likely plays a significant role. This suggests that PARPi induces H3K9me2-associated replication stress, thereby increasing ATR dependence for fork progression. DNA damage was then measured by monitoring γH2A.X levels. Either niraparib or tuvusertib alone resulted in a limited increase in γH2A.X, whereas their combination further increased γH2A.X in S-phase cells, which was significantly attenuated by UNC0642 (Fig. 3H). Accordingly, UNC0642 partially but significantly rescued the viability of cells treated with the tuvusertib/niraparib combination, but not each alone (Fig. 3I). These findings suggested that an increase in H3K9me2-associated heterochromatin induced by PARP inhibition at least partially confers susceptibility to ATR inhibition.

### SMARCA4 deficiency exacerbates ATRi/PARPi-induced DNA damage through Mre11 and Mus81 nucleases

**Fig. 4:**
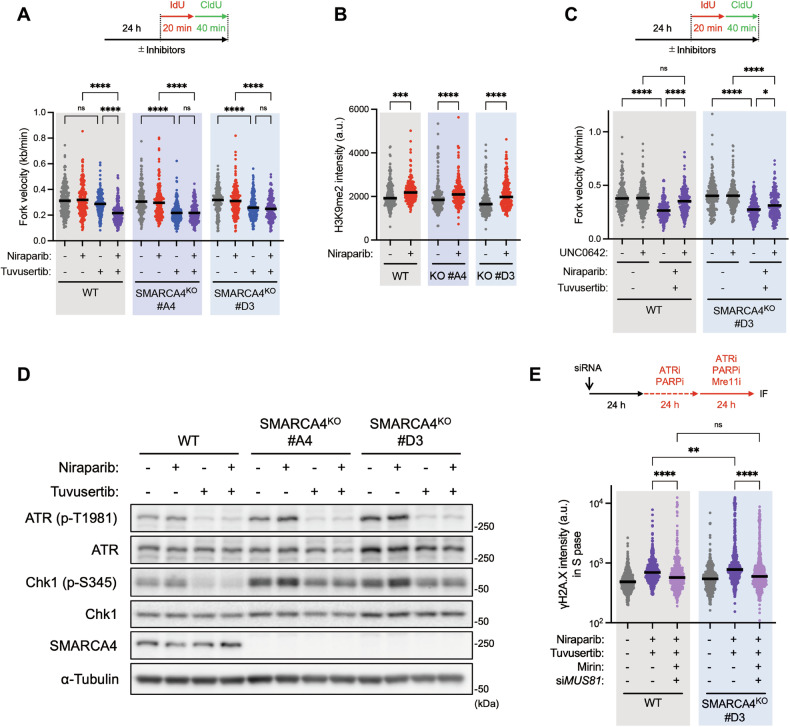
SMARCA4 deficiency exacerbates Mus81/Mre11-mediated DNA damage upon ATR/PARP inhibition. **A** Dot plot of fork velocity in SMARCA4^WT^ and SMARCA4^KO^ H1975 cells. Cells were treated with 10 nM tuvusertib and 625 nM niraparib for 24 h prior to IdU and CldU labeling, then a DNA fiber assay was performed. The graph indicates the mean of 200 fibers and statistical significance by Kruskal–Wallis test with Dunn’s multiple comparisons test. *P*-value: not significant (ns) > 0.05 and **** ≤ 0.0001. **B** Dot plot of H3K9me2 mean intensity per nuclei in SMARCA4^WT^ and SMARCA4^KO^ H1975 cells. Cells were treated with 2.5 µM niraparib for 24 h, then were carried on immunofluorescence analysis. The graph indicates the median of 200 cells and statistical significance by Kruskal–Wallis test with Dunn’s multiple comparisons test. **C** Dot plot of fork velocity in SMARCA4^WT^ and SMARCA4^KO^ H1975 cells. Cells were treated with 10 nM tuvusertib, 625 nM niraparib, and 2 µM UNC0642 for 24 h prior to IdU and CldU labeling, then a DNA fiber assay was performed. The graph indicates the mean of 200 fibers and statistical significance by Kruskal–Wallis test with Dunn’s multiple comparisons test. *P*-value: not significant (ns) > 0.05, * ≤ 0.05, and **** ≤ 0.0001. **D** Western blot analysis of SMARCA4^WT^ and SMARCA4^KO^ cells. Cells were treated with 62.5 nM tuvusertib and 625 nM niraparib for 48 h, then carried on western blot analysis. α-Tubulin was detected as loading control. **E** Dot plot of γH2A.X mean intensity per nuclei in S phase cells. Cells were transfected with siRNA for 24 h, then were treated with 62.5 nM tuvusertib and 625 nM niraparib for 48 h and 25 µM Mirin for 24 h prior to immunofluorescence analysis. PCNA-positive cells were defined as S phase cells. The graph indicates the median of 500 cells and statistical significance by Kruskal–Wallis test with Dunn’s multiple comparisons test. *P*-value: ** ≤ 0.01 and **** ≤ 0.0001.

Next, we examined the mechanism by which the combined use of ATRi and PARPi in SMARCA4^KO^ cells increased their sensitivity compared to SMARCA4^WT^ cells. SWI/SNF complexes, in which SMARCA4 is a constitutive factor, oppose polycomb repressive complexes (PRCs) via ATP-dependent eviction of nucleosomes, leading to the formation of accessible chromatin [50]. Reversal of this process, such as that resulting from the loss of SMARCA4, leads to the reassembly of facultative heterochromatin [51]. Furthermore, we and others have reported that loss of SMARCA4 drives HP1/H3K9me3-mediated heterochromatin formation [52], resulting in increased RS and higher susceptibility to ATR inhibition [21]. Consistently, in our experimental models, ATR and Chk1 were also activated in unchallenged SMARCA4^KO^ cells compared with SMARCA4^WT^ cells (Fig. S4A). Therefore, we hypothesized that SMARCA4 deficiency-associated heterochromatin formation may be an additional source of RS that reinforces PARP inhibition-induced heterochromatin-associated RS. We analyzed whether ATR inhibition with or without PARP inhibition impaired fork progression in SMARCA4-deficient cells. We found that tuvusertib alone significantly reduced fork velocity in SMARCA4^KO^ cells, but not in SMARCA4^WT^ cells, suggesting that SMARCA4-deficient cells depend on ATR activity for fork progression (Figs. 4A, S4A). With ATRi, forks may regress under the action of fork remodeling factors like SMARCAL1 [7], and the regressed arms become targets for Mre11 nuclease [21], leading to moderate but significant cell death in SMARCA4^KO^ cells compared to SMARCA4^WT^ cells (Fig. 2C). Importantly, while the fork velocity of SMARCA4^WT^ cells was reduced by the tuvusertib/niraparib combination potentially due to increased heterochromatin, no further reduction in fork velocity was observed in SMARCA4^KO^ cells compared to tuvusertib alone (Fig. 4A). However, niraparib may further exacerbates the situation in SMARCA4^KO^ cells by promoting the accumulation of H3K9me2-associated heterochromatin (Fig. 4B), as evidenced by the partial rescue of reduced fork progression by UNC0642 (Fig. 4C). We noticed that niraparib-treated SMARCA4^KO^ cells activated ATR and Chk1 more strongly than SMARCA4^WT^ cells, suggesting an increased dependency of these cells to ATR for fork protection and cell survival (Fig. 4D). These results, along with an increase in DNA damage induced by tuvusertib/niraparib combination (Fig. 2F), prompted us to test if fork stability would be compromised in SMARCA4^KO^ cells when ATR and PARP activity were simultaneously inhibited. Several replication-coupled repair mechanisms are used to promote the restart of stalled forks. Stalled forks can be reversed by the action of DNA translocases such as ZRANB3, HLTF, and SMARCAL1 and recombinase Rad51 [2]. The resulting four-way structure can be cleaved by nucleases such as Mus81 to form DSBs, which activate ATR to promote fork restart and to limit excessive cleavage of reversed forks by Mus81, indicating that a MUS81-triggered and ATR-mediated feedback loop finetunes Mus81 activity at replication forks [53]. However, when ATR activity is inhibited, DSBs induced by Mus81 accumulate at high levels and can also be toxic to cells [53]. Alternatively, defects in fork protection factors such as BRCA1/2 and Rad51 cause Mre11 nuclease-mediated degradation and/or cleavage at stalled forks, thereby sensitizing cells to RS-inducing agents [54, 55]. We previously reported that SMARCA4 loss causes ATRi-induced ssDNA exposure via Mre11-dependent degradation of nascent DNA at reversed forks [21]. In our model, Mre11 inhibition with mirin was not able to suppress the increase in tuvusertib/niraparib-induced DNA damage in SMARCA4^KO^ cells (Fig. S4B), speculating that Mus81 could cleave intact forks (without nicks) [56] and other nucleases, besides Mre11, might contribute to degrade nascent DNA. In contrast, Mus81 depletion with siRNA further enhanced tuvusertib/niraparib-induced DNA damage, possibly because excessive reversed forks were attacked by Mre11 in the presence of tuvusertib (Fig. S4C, D). Notably, Mre11 inhibition in Mus81-depleted SMARCA4^KO^ cells significantly suppressed the increase in tuvusertib/niraparib-induced DNA damage, suggesting that stalled forks at heterochromatin caused by SMARCA4 loss and PARP inhibition may be attacked synergistically by both Mre11 and Mus81 when the ATR/Chk1 pathway is inhibited (Fig. 4D, E) [57], leading to massive fork collapse. This is consistent with a previous report in which Mus81 was thought to depend on nicking by MRE11 before incising DNA [58]. Importantly, the effects of both nucleases were significant but weaker in SMARCA4^WT^ cells compared to SMARCA4^KO^ cells (Fig. 4E), suggesting that SMARCA4 protects cells from excessive nuclease processing upon ATR/PARP inhibition. Taken together, these results indicated that PARP inhibition in SMARCA4-deficient cells increased RS via amplification of heterochromatin, and ATR inhibition under these conditions exacerbated DNA damage by attacking genomic DNA with Mre11 and Mus81 nucleases, resulting in fork collapse and severe cell death.

### ATRi/PARPi combo-therapy suppresses tumor growth in SMARCA4-deficient LUAD xenograft models

**Fig. 5:**
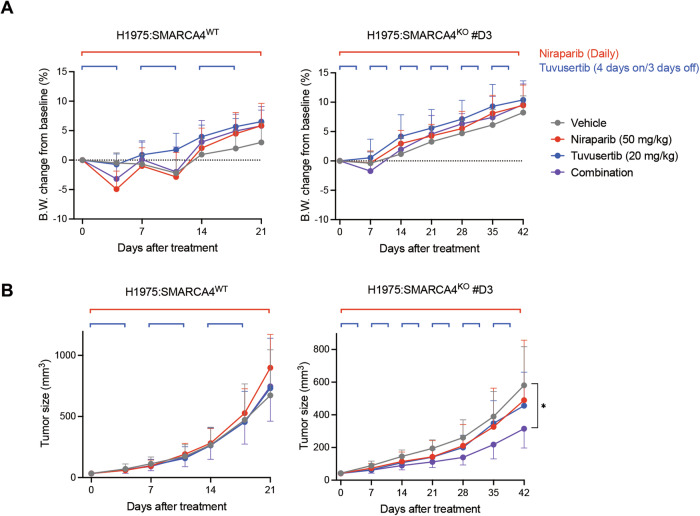
ATRi/PARPi combo-therapy is more effective than ATRi alone in SMARCA4-deficient LUAD xenograft models. **A, B** Body weight (B.W.) changes relative to day 0 and tumor growth in each group. H1975:SMARCA4^WT^ and SMARCA4^KO^ xenograft mice were treated with niraparib (50 mg/kg, Daily), tuvusertib (20 mg/kg, 4 days on/3 days off), or combination. The graph indicates the mean ± SD of *n* = 10 mice/group and statistical significance by two-way ANOVA test with Tukey’s multiple comparisons test. *P*-value: * ≤ 0.05.

To test whether in vitro combinations of tuvusertib/niraparib in SMARCA4-deficient tumors translated in vivo and to provide insights into optimal dose-schedules, that balance tolerability and efficacy, we established xenograft models subcutaneously implanted with H1975:SMARCA4^WT^ or SMARCA4^KO^ cells in BALB/c nude mice. Despite the proven synergy, the challenge of ATRi/PARPi combo-therapy is the increased incidence of adverse events such as hematologic toxicity in preclinical and clinical settings [59]. We, therefore, used continuous niraparib (50 mg/kg, daily) and/or intermittent tuvusertib (20 mg/kg, 4 days on/3 days off) by oral administration in mice as either monotherapy or combo-therapy, and these tolerability and anti-tumor effects were assessed. In both tumor models, all treatments were well tolerated, with no mice losing ≥ 10% body weight and showing no significant decrease in body weight compared to the vehicle group during the study period (Fig. 5A). In SMARCA4^WT^ xenograft models, all treatments, including combo-therapy, had little effect on tumor growth compared to the vehicle group (Fig. 5B). Notably, in SMARCA4^KO^ xenograft models, tuvusertib or niraparib monotherapy weakly suppressed tumor growth compared to that in the vehicle group, but this was not statistically significant (Fig. 5B). More importantly, tuvusertib/niraparib combo-therapy significantly suppressed SMARCA4^KO^ tumor growth compared to the vehicle group and exhibited stronger antitumor activity than the respective monotherapy (Fig. 5B). These results are consistent with the in vitro findings shown in Fig. 2C, suggesting that the use of PARPi as a combined drug with ATRi and SMARCA4 deficiency as a genetic biomarker are critical determinants for exhibiting potent antitumor activity of ATRi. Together, our pre-clinical study provides a rationale for the clinical development of ATRi/PARPi combo-therapy targeting SMARCA4-deficient LUAD.

## Discussion

**Fig. 6:**
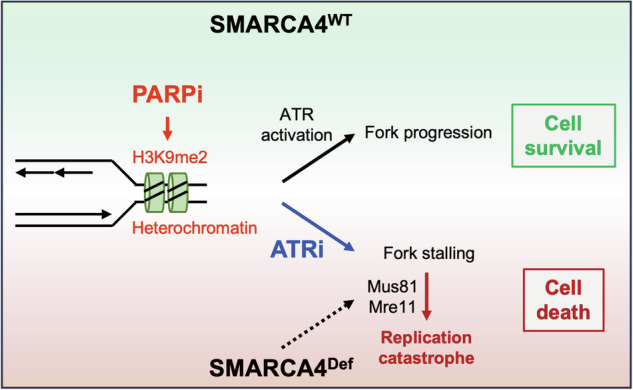
Graphic summary of action of ATRi/PARPi combination in SMARCA4-deficient cells. PARP inhibition elevates H3K9me2-associated heterochromatin that increases ATR dependence for fork progression and cell survival, resulting in moderate synergistic cytotoxicity with ATR inhibition in SMARCA4 wild-type (WT) cells. In SMARCA4-deficient (Def) cells, Mus81 and Mre11 nucleases cooperate to exacerbate ATRi/PARPi-induced replication catastrophe. Therefore, ATRi and PARPi combination causes potent cytotoxicity in SMARCA4-deficient cells.

Since ATR kinase is an essential responder to tolerate harmful RS in cancer, ATRis have been developed as a potent and selective anti-cancer drug in a series of pre-clinical studies and clinical trials [3, 17, 18]. A promising approach for ATRi therapy is its use in combination with other drugs and/or biomarkers that increase RS levels or disrupt the DDR pathways. Even though the current phase I/II studies have observed several patients who received ATRi-based combination therapies, achieving complete or partial responses [60–63], there is a need to improve the therapeutic approach by considering both effective combined drugs and beneficial sensitivity biomarkers for successful clinical development. In this study, we performed drug combination screening using LUAD cell lines harboring loss of function of SWI/SNF-complex components and determined PARPi (niraparib) as an effective combined drug with ATRi (tuvusertib) and SMARCA4 deficiency as a beneficial sensitivity biomarker for this combination. Mechanistically, (i) the enhancement of heterochromatin-associated RS by PARP activity inhibition increases ATR dependence to maintain fork stability and (ii) inhibition of ATR activity in SMARCA4-deficient cells increases Mre11- and Mus81-mediated DNA damage in stressed forks, indicating an increased ATRi susceptibility of SMARCA4 deficient LUAD cells (Fig. 6). Tuvusertib/niraparib combo-therapy demonstrated greater anti-tumor activity than tuvusertib monotherapy in SMARCA4-deficient LUAD xenograft models. Since SMARCA4 mutations are present in approximately 7-8% of LUAD patients [64, 65], the ATRi/PARPi combination may offer a therapeutic benefit for those with SMARCA4-deficient tumors.

SWI/SNF complexes are ATP-dependent chromatin remodelers that modulate genomic architecture and DNA accessibility, enabling precise and timely control of gene expression [19]. Recent research has clarified that the 29 genes encoding SWI/SNF subunits form three distinct SWI/SNF complexes: canonical BAF (cBAF), polybromo-associated BAF (PBAF), and noncanonical BAF (ncBAF), each containing both shared and complex-specific subunits. SMARCA4, which possesses DNA-stimulated ATPase activity, is present in all three complexes, while ARID1A is specific to the cBAF complex, and PBRM1 is unique to the PBAF complex. Our screening of LUAD cell lines harboring loss of function of SWI/SNF components revealed that the loss of SMARCA4 is a genetic biomarker strongly associated with forced ATRi- and ATRi/PARPi-induced cytotoxicity, consistent with our previous results, whereas neither ARID1A nor PBRM1 defects were unexpectedly not, suggesting that ATRi and ATRi/PARPi-induced cytotoxicity may require defects of all three complexes rather than partial defects. Moreover, SWI/SNF complexes are recruited to damaged chromatin via direct interactions between SMARCA4, H2A.X nucleosomes and acetylated histone H3 [66], contributing functionally important roles for both non-homologous end joining (NHEJ) and homologous recombination (HR) [67]. Besides, SMARCA4 recruits topoisomerases to chromatin [68, 69], and its loss may lead to DNA replication stress. Moreover, in an ARID1A-mutant LUAD cells, deletion of SMARCA4 increased sensitivity to ATR inhibitors, suggesting that SMARCA4 and ARID1A function in parallel [22]. Thus, the loss of SMARCA4 could impair DNA repair and enhance intrinsic replication stress through multiple mechanisms, possibly explaining the specific association between SMARCA4 deficiency and ATR inhibitor efficacy in LUAD cells, although we do not exclude possibilities that loss of ARID1A or PBRM1 could also serve as biomarkers for ATRi-based therapy in other cancer types. Notably, recent RNAi screens identified ARID1A and PBRM1 as genetic determinants of ATRi sensitivity, although these screens did not hit SMARCA4, suggesting that constitutive loss, but not acute loss, of SMARCA4 renders cells susceptible to ATRi. When SWI/SNF function is compromised by loss of SMARCA4, chromatin becomes more condensed, leading to the formation of heterochromatin [70], which is usually difficult to replicate and can be an obstacle to fork progression [2]. During the adaptation process to the loss of SMARCA4, cells might become tolerant to heterochromatin-associated RS through ATR-dependent replication fork regulation [21]. Moreover, we reported that oncogenic KRAS-induced heterochromatin-associated RS was tolerated in an ATR-dependent manner [14]. These findings suggest that heterochromatin-associated RS increases dependence on ATR activity and vulnerability to ATRis.

Our results showed that PARPi-treated cells accumulated H3K9me2-associated heterochromatin, making replication forks rely on ATR activity for progression. Pharmacological H3K9me2 silencing by UNC0642 significantly recovered ATRi-induced fork slowing, suggesting that PARP inhibition-induced heterochromatin is a key source of RS that increases sensitivity to ATRi. PARP has been involved in not only DNA repair but also chromatin remodeling [49]. Accumulating evidence indicates that PARP causes the relaxation of chromatin structure via PARylation of core histones [71, 72] and that PARP activity regulates the recruitment of chromatin remodelers ALC1 and SMARCA5 and the stability of histone modifier EZH2 [73–76], suggesting that PARP inhibition potentiates heterochromatin formation through multiple mechanisms. Notably, a recent study revealed that PARPi impedes the resolution of RS induced by heterochromatin associated with H3K9me2 in oncogenic IDH1/2-mutant cells [48], which supports our finding that PARPi increases the level of H3K9me2. Moreover, the reduction of heterochromatin by UNC0642 likely modulates the expression of many genes [77]. However, Gaggioli and colleagues reported that almost no anomalous expression was observed for a large set of DDR genes in UNC0642-treated conditions [78]. They suggested that G9a’s function in regulating the chromatin landscape at replication forks is unrelated to its role in transcriptional regulation. In the same report, they observed that UNC0642 reduces the levels of fork remodelers like Rad51 and HLTF at stalled forks, which may decrease fork reversal and promote fork progression. These would also contribute the rescuing effects of UNC0642 for increased ATRi sensitivity with PARPi. Alternatively, PARP trapping on DNA and/or transcription-replication conflicts, both resulting from inhibition of the catalytic activity of PARPs, might cause RS [29, 79]. Although the mechanism by which PARP activity resolves RS and its inhibition propagates RS still requires further investigation, our results provide a previously unrecognized mechanism of action of ATRi under PARPi-induced RS.

How is replication fork progression maintained at around the heterochromatic genomic region? We found that ATR activity is required for sustaining fork progression under PARP inhibition-induced heterochromatin-associated RS in SMARCA4^WT^ cells and in SMARCA4^KO^ cells even in the absence of PARPi, raising the possibility that ATR may drive alternative polymerases to avoid prolonged fork stalling. PARP inhibition inhibits the repair of unligated Okazaki fragments [80], potentially leaving ssDNA gaps on the lagging strand [45] and suppresses fork reversal by allowing RECQ1 to resolve reversed forks [81], This may increase the reliance on PrimPol at stressed forks and lead to the formation of PrimPol-generated ssDNA gaps on the leading strand [9]. Furthermore, we and others previously reported that PrimPol is phosphorylated at Ser255 in an ATR/Chk1 dependent manner in response to RS, thereby promoting its repriming activity [82] and overcoming RS challenges such as heterochromatin regions [14]. However, PrimPol activity might be compromised in the presence of ATRi, hindering fork progression and exacerbating fork regression. Thus, these findings suggest a possible mechanism underlying the synergistic effects of the ATRi/PARPi combination in SMARCA4^WT^ cells. In SMARCA4^KO^ cells, however, ATR inhibition itself reduced fork velocity in the absence of PARPi due to RS associated with SMARCA4 loss, and ATR/PARP inhibition did not further reduce fork velocity, followed by exacerbated DNA damage dependent on the simultaneous activation of Mre11 and Mus81 nucleases. While a reduced replication fork speed is often linked to cellular fitness and DNA damage, it does not always directly correlate with decreased cell viability. Rather, fork status, which is influenced by factors such as fork protection and remodeling, may have a greater impact on DNA damage and cell viability. Recent studies have revealed that homologous recombination (HR) factors are required to protect stalled/reversed forks from excessive degradation by nucleases [83, 84]. Furthermore, SMARCA4 contributes to HR by interacting with BRCA1 and promoting Rad51 loading on ssDNA [85, 86]. ATR also regulates the functions of Rad51 and its paralog complexes and fine-tunes the activity of Mus81 nuclease under RS [11, 53, 87, 88]. Therefore, under synergistically severe RS under SMARCA4 loss and PARP inhibition, cells may highly rely on ATR-dependent mechanisms for fork protection. Furthermore, holiday junction-like structures at reversed forks may become more susceptible to Mus81-mediated cleavage due to inefficient SLX4 recruitment caused by impaired PARylation in the presence of PARPi [89]. This, in turn, can lead to Mre11-dependent degradation of nascent DNA, resulting in increased DNA damage and fork collapse by ATR inhibition. Thus, our findings imply that addiction to ATR for fork protection and sustainable fork progression is closely associated with vulnerability to ATRis in cancer cells.

In the past decade, ATRi/PARPi combo-therapy has been tested as a promising approach in clinical trials based on in vitro evidence that PARPis cause cellular stress that requires the activation of ATR signaling for cell survival. Yet, these clinical studies showed limiting responders following treatment with the ATRi/PARPi combination [90, 91]. Our data indicate that in H1975:SMARCA4^WT^ cells, in vitro treatment with the ATRi/PARPi combination exhibited potent synergistic cytotoxicity at relatively high concentrations, whereas in vivo activity of the combination had little effect on their tumor growth. Since high doses of ATRi and PARPi result in an increased incidence of side effects such as hematological toxicity, targeting tumors exhibiting biomarkers that confer a synergistic effect at their bioavailable low doses is crucial for successful antitumor activity. In agreement with this idea, the ATRi/PARPi combination reduced in vitro cell viability at low concentrations in H1975:SMARCA4^KO^ cells compared to SMARCA4^WT^ cells and exhibited significant anti-tumor activity in H1975:SMARCA4^KO^ xenograft models. Therefore, genetic defects, including SMARCA4 in our study, and ATM or RNase H2 defects recently reported by others [31, 92], can be beneficial biomarkers for ATRi/PARPi combo-therapy. Interestingly, ATRis have been reported to promote anti-tumor immune responses in cooperation with ATRi-induced DNA damage in tumor cells [93–95]. Furthermore, rapid immune cell recovery and complete response after combo-therapy with anti-PD-L1 requires intermittent rather than daily ATRi treatment [96]. Our in vivo experiments were also tested by intermittent treatment with ATRi, and anti-tumor activity was observed even in immune-deficient models. In the future, testing a tolerable and effective dosing schedule of ATRi and PARPi in immune-competent models could provide an opportunity to improve prognosis through innate immune response or immunotherapy.

Overall, our results provide previously unknown insights into the mechanism of action of combo-therapy, in which heterochromatin-associated RS induced by PARPi acts synergistically with ATRi and highlight that intermittent ATRi and continuous PARPi combination targeting SMARCA4-deficient LUAD could be an attractive approach for successful ATRi therapy.

## Methods

### Cell lines and cell culture

The LUAD cell lines used in this study are described in Table S1. A549 and HEK293T (ATCC) cells were cultured in Dulbecco’s modified Eagle’s medium (Nacalai Tesque, #08459-64) supplemented with 10% fetal bovine serum (FBS) (Gibco, A5256701) and penicillin-streptomycin (Nacalai Tesque, #26252-94). H1299, H2172, SK-LU-1, ABC1, H226, and H1975 cells were cultured in RPMI 1640 medium (Nacalai Tesque, #30264-56) supplemented with 10% FBS and penicillin-streptomycin. All the cells were cultured in a 5% CO_2_ incubator at 37 °C.

To establish SMARCA4-, ARID1A-, and PBRM1-restoring cells, we used the lentiviral cDNA expression vector, CSII-CMV-MCS-IRES2-Bsd (RIKEN, RDB04385). The SMARCA4 expression vector was prepared as described previously [21]. Full-length ARID1A cDNA was amplified by polymerase-chain reaction (PCR) using the primers 5’-CGTCAGATCCGCTAGCCACCATGGCCGCGCAGGT-3’ and 5’-GGCGGATCCGCGGCCGCTCATGACTGGCCAATCAAAAACAGTA-3’ and pLenti-puro-ARID1A (Addgene, #39478) as template DNA. Full-length PBRM1 cDNA was amplified by PCR using the primers 5’- CGTCAGATCCGCTAGCCACCATGGGTTCCAAGAGAAGAAG-3’ and 5’- GGCGGATCCGCGGCCGCTTAAACATTTTCTAGGTTGT-3’ and TetO-FUW-PBRM1-pgk-puro (Addgene, #85746) as template DNA. These cDNA products were inserted into NheI/NotI-digested CSII-CMV-MCS-IRES2-Bsd using an In-Fusion HD cloning kit (Clontech, #639649). To produce the lentivirus, HEK293T cells were co-transfected with pCAG-HIVgp (RIKEN, RDB04394), pCMV-VSV-G-RSV-Rev (RIKEN, RDB04393), and SMARCA4, ARID1A, or PBRM1 expression vectors using polyethylenimine “MAX” (Polysciences, #24765). Forty-eight hours later, the lentivirus-containing medium was collected and concentrated using a Lenti-X concentrator (Clontech, #631232), according to the manufacturer’s protocol. To establish SMARCA4-, ARID1A-, and PBRM1-restroing cells, SMARCA4-deficient cells (A549 and H1299), ARID1A-deficient cells (H2172 and SK-LU-1), and PBRM1-deficient cells (ABC1 and H226) were infected with each corresponding lentivirus in appropriate medium supplemented with 8 µg/mL polybrene (Sigma-Aldrich, H9268) for 24 h. These cells were cultured in appropriate medium supplemented with 10 µg/mL blasticidin S (FUJIFILM Wako Pure Chemical Co, #022-18713).

To establish SMARCA4 knockout cells, we used the human Cas9/sgRNA expression vector pSpCas9(BB)-2A-Puro (PX459) V2.0 (Addgene, #62988). pSpCas9(BB)-2A-Puro was digested with BbsI and dephosphorylated by shrimp alkaline phosphatase (New England Biolabs, M0371L). The DNA insert for SMARCA4-targeting sgRNA was prepared by annealing oligo DNA 5’-CACCGGGTCCTGTTGCGGACACCG-3’ and 5’-AAACCGGTGTCCGCAACAGGACCC-3’. The DNA insert was phosphorylated by T4 polynucleotide kinase (New England Biolabs, M0201L). The BbsI-digested pSpCas9(BB)-2A-Puro was ligated with the DNA insert using a Quick Ligation kit (New England Biolabs, M2200L). To establish SMARCA4 knockout cells, H1975 cells were transfected with the Cas9/sgRNA expression vector using Lipofectamine 3000 transfection reagent (Invitrogen, L3000015). The cells were cultured in the appropriate medium supplemented with 1 µg/mL puromycin (InvivoGen, ant-pr-1) for 24 h and carried to single-cell cloning.

### Drugs

ATRi (tuvusertib), ATMi (lartesertib), DNA-PKi (peposertib), and PARPi (niraparib) were provided by the healthcare business of Merck KGaA, Darmstadt, Germany (CrossRef Funder ID: 10.13039/100009945). Camptothecin (CPT) was purchased from Abcam (ab120115). G9a/GLPi (UNC0642) was purchased from Selleck Chemicals (S7230). Mre11i (mirin) was purchased from Sigma-Aldrich (M9948). All compound stocks were diluted in DMSO for in vitro experiments.

### siRNA transfection

Cells were reverse-transfected with siRNA at using Lipofectamine RNAiMAX transfection reagent (Invitrogen, #13778150), according to the manufacturer’s protocol with minor modifications. We used MISSION siRNA Universal Negative Control #2 (siNC, Sigma-Aldrich, SIC002) and siPARP1 (5’-AAGAUAGAGCGUGAAGGCGAAtt-5’) at final concentration of 2.5 nM in Figs. 3E, S3B [97]. We used silencer select siRNAs (Ambion); negative control #1 siRNA (siNC, #4390843) and siMUS81 (s37038) at final concentration of 1 nM in Figs. 4E, S4C, D.

### Cell viability assay

Cells were seeded in 96-well white plates. Twenty-four hours later, the cells were treated with drugs and cultured for 4-6 days. Cell viability was assessed using the CellTiter-Glo 2.0 cell viability assay reagent (Promega, G9243), according to the manufacturer’s protocol with minor modifications. Luminescent signals were detected on a microplate reader Synergy H1 (BioTek). We used the SynergyFinder Plus to analyze the drug combination matrix data.

### Western blotting

Cells were directly lysed in 2 × SDS sample loading buffer (125 mM Tris-HCl pH6.8, 4% SDS, 0.01% bromophenol blue, 20% Glycerol, 200 mM dithiothreitol in water) and denatured at 95 °C for 10 min. Protein concentrations in the cell lysates were estimated using XL-Bradford (APRO Science, KY-1031). Equal amounts of protein were separated by SDS-PAGE at 100 V for approximately 2 h and then transferred to polyvinylidene difluoride (PVDF) membranes (Millipore, IPVH00010) at 90 mA at 4 °C for 12-16 h. The membranes were blocked with 5% skim milk/TBS-T (25 mM Tris-HCl pH7.8, 140 mM NaCl, 0.1% Tween 20 in water) or Blocking One-P (Nacalai Tesque, #05999-84) at room temperature for 30 min, then incubated with the following primary antibodies; anti-SMARCA4 (1:1000, Cell Signaling Technology, #49360), anti-ARID1A (1:1000, Cell Signaling Technology, #12354), anti-PBRM1 (1:1000, Cell Signaling Technology, #91894), anti-ATR p-T1989 (1:1000, Cell Signaling Technology, #30632), anti-ATR (1:1000, Cell Signaling Technology, #13934), anti-Chk1 p-S345 (1:1000, Cell Signaling Technology, #2348), anti-Chk1 (1:500, Santa Cruze Biotechnology, sc-8408), anti-ATM p-S1981 (1:1000, Cell Signaling Technology, #5883), anti-ATM (1:1000, Cell Signaling Technology, #2873), anti-Chk2 p-T68 (1:1000, Cell Signaling Technology, #2197), anti-DNA-PK p-S2056 (1:1000, Abcam, ab124918), anti-DNA-PK (1:1000, Cell Signaling Technology, #38168), RPA32 p-S4/8 (Bethyl laboratories, A300-245A), anti-Poly/Mono-ADP-Ribose (1:1000, Cell Signaling Technology, #83732), anti-PARP (1:1000, Cell Signaling Technology, #9532), anti-Mus81 (1:1000, Abcam, ab14387), anti-β-Actin (1:5000, Sigma-Aldrich, A5441), and α-Tubulin (1:5000, Medical & Biological laboratories, PM054) diluted in blocking buffer at room temperature for more than 4 h or at 4 °C overnight. After three washes with TBS-T for 5 min each, the membranes were incubated with the following secondary antibodies; Peroxidase AffiniPure Goat Anti-Mouse IgG (H + L) (1:5000, Jackson ImmunoResearch Laboratories, 115-035-003) and Peroxidase AffiniPure Goat Anti-Rabbit IgG (H + L) (1:5000, Jackson ImmunoResearch Laboratories, 111-035-003) diluted in blocking buffer at room temperature for 1 h. After three washes with TBS-T for 10 min each, chemiluminescence signals were detected using Western Lightning Plus-ECL (PerkinElmer, NEL105001EA) or ImmunoStar Zeta (FUJIFILM Wako Pure Chemical Co, #295-72404) and imaged on an ImageQuant LAS 3000 luminescent image analyzer (FujiFilm) or FUSION SOLO.7S.EDGE chemiluminescence imaging system (Vilber Bio Imaging). The original western blots were shown in Supplementary Material.

### Immunofluorescence

Cells were seeded in 8-well chamber slides. For immunostaining, the cells were pre-fixed on ice for 5 min and then pre-extracted with 0.5% Triton X-100/CSK100 (100 mM NaCl, 10 mM MOPS pH7.0, 3 mM MgCl_2_, 300 mM sucrose in water) for 5 min on ice. The cells were fixed with 4% PFA/CSK100 at room temperature for 10 min and then permeabilized with 0.5% Triton X-100/CSK100 at room temperature for 10 min. For experiments involving PCNA staining, cells were fixed with 4% PFA/CSK100 for 10 min and then fixed and permeabilized with 100% methanol at −20 °C for 10 min. After washing with 3% BSA/PBS-T (0.05% Tween 20 in PBS), the slides were blocked with 3% BSA/PBS-T at room temperature for 30 min, and then incubated with the following primary antibodies; anti-PCNA (1:500, Cell Signaling Technology, #2586), anti-γH2A.X (1:500, Cell Signaling Technology, #9718), anti-H3K9me2 (1:500, Abcam, ab1220), and anti-PARP (1:500, Cell Signaling Technology, #9532) diluted in blocking buffer at 37°C for 1 h. After three washes with 3% BSA/PBS-T, the slides were incubated with the following secondary antibodies; Alexa Fluor 488-AffiniPure Donkey Anti-Mouse IgG (H + L) (1:500, Jackson ImmunoResearch Laboratories, 564-786019) and Alexa Fluor 594-AffiniPure Donkey Anti-Rabbit IgG (H + L) (1:500, Jackson ImmunoResearch Laboratories, 561-78471) diluted in blocking buffer at room temperature for 1 h. Nuclei were stained with 1 µg/mL DAPI in PBS-T at room temperature for 5 min. After three washes with PBS-T, the slides were placed in PBS and mounted with VECTASHIELD PLUS Antifade Mounting Medium (Vector Laboratories, H-1900). Images and fluorescence intensities were acquired on a fluorescence microscope Celldiscoverer 7 (Zeiss).

### Proximity ligation assay (PLA) for in situ analysis of protein interaction with nascent DNA replication forks

Cells were seeded in 8-well chamber slides. S-phase cells were pulse labeled with 10 μM EdU (Invitrogen, A10044) for 30 min. As mentioned in the immunofluorescence method, cells were pre-extracted, fixed, permeabilized and processed with Click-iT Cell Reaction Buffer Kit (Invitrogen, C10269) and biotin azide (Invitrogen, B10184), according to the manufacturer’s protocol. After washing with 3% BSA/PBS-T (0.05% Tween 20 in PBS), the slides were blocked with 3% BSA/PBS-T at room temperature for 30 min, and then incubated with the following primary antibodies; anti-Biotin (1:2500, Jackson ImmunoResearch, #200-002-211) and anti-γH2A.X (1:1000, Cell Signaling Technology, #9718) diluted in blocking buffer at 4 °C overnight. PLA was performed using Duolink In Situ PLA kits (Sigma-Aldrich, DUO92001, DUO92005, DUO92008), according to the manufacturer’s protocol. Nuclei were stained with 1 µg/mL DAPI in PBS at room temperature for 5 min. After three washes with PBS, the slides were mounted with VECTASHIELD PLUS Antifade Mounting Medium (Vector Laboratories, H-1900). Images and number of foci were acquired on a fluorescence microscope Celldiscoverer 7 (Zeiss).

### DNA fiber assay

Cells were pulse-labeled with 50 µM IdU for 20 min, washed twice with PBS, and then pulse-labeled with 250 µM CldU for 40 min. Labeled cells were harvested by trypsinization and fixed with a freshly prepared fixative solution of methanol:acetic acid (3:1). The fixed cells were pelleted at 800 × g for 5 min at 4°C, resuspended in fixative solution at a final concentration of 1000-2000 cells/µL, and then mixed with a 5-10-fold excess of unlabeled cell suspension. 2-3 drops of the cell suspension were spotted onto slides. After drying, the slides were immersed in lysis buffer (200 mM Tris-HCl pH 7.5, 50 mM EDTA, 0.5% SDS in water) at 37 °C for 15-20 min. The slides were tilted at an appropriately 30° angle to spread the DNA fibers at a low speed. After drying, the slides were immersed in a fixative solution for 20 min and dried at 4 °C overnight.

For the immunostaining of DNA fibers, the DNA fibers were rehydrated in PBS twice for 5 min and then immersed in 2.5 M HCl for 1 h to denature the DNA. After three washes with PBS for 5 min each, the slides were blocked with 5% BSA/PBS-T (0.1% Tween 20 in PBS) at 37 °C for 1 h and incubated with anti-IdU (1:25, BD Biosciences, 347580) and anti-CldU (1:100, Abcam, ab6326) antibodies diluted in blocking buffer at 37 °C for 2 h. After three washes with PBS-T for 5 min each, the slides were incubated with anti-rat IgG conjugated with Alexa Fluor 488 (1:500, Invitrogen, A11006) and anti-mouse IgG conjugated with Alexa Fluor 555 (1:100, Invitrogen, A21422) diluted in blocking buffer at room temperature for 1 h. After three washes with PBS-T for 5 min each, the slides were placed in PBS and mounted with ProLong Gold Antifade (Invitrogen, P36980). Images were acquired on a fluorescence microscope Celldiscoverer 7. Using ImageJ software (National Institutes of Health), we measured the fiber length of only contiguous IdU-CldU tracts which characterize progressing replication forks. Finally, 1 µm of fiber length was converted to 2.59 kb DNA.

### Animal experiment

H1975:SMARCA4^WT^ or H1975:SMARCA4^KO^ #D3 cells were subcutaneously injected at 1 × 10^6^ cells/mouse suspended in 100 µL of 1:1 PBS(-):matrix (Gibco, A1413202) into the right flank of 6 week-old female BALB/c-nu/nu mice (The Jackson Laboratory Japan). When tumor size reached at 20–80 mm^3^, mice were randomized into vehicle, niraparib monotherapy, tuvusertib monotherapy, or tuvusertib/niraparib combo-therapy group, then were started on treatment. Niraparib was diluted in 0.5% METHOCEL/0.25% Tween 20 in water, and tuvusertib was diluted in 15% Captisol in water. The mice were orally administered vehicle (0.5% METHOCEL/0.25% Tween 20, daily), niraparib (50 mg/kg, daily), or tuvusertib (20 mg/kg, 4 days on/3days off). In the tuvusertib monotherapy group, mice were administered vehicle during the cessation period. In the combo-therapy group, mice were administered niraparib first, followed by tuvusertib 30-45 min later. Body weight changes were calculated relative to the individuals’ body weight on day 0. Tumor size was calculated using the following formula: (long length x short wide^2^)/2. All animal experiments were performed in accordance with relevant guidelines and regulations and were approved by the National Cancer Center Research Institute of Laboratory Animal Research (T21-011-M02). Endpoints were determined based on ≥10% body weight loss or exceeding tumor volume of ≥10% of body weight. Tumor volume was converted into tumor weight (i.e. 1000 mm^3^ corresponds to 1 g). All experiment was terminated before all mice reached these criteria.

### Statistical analysis

All statistical analyses were performed using the GraphPad Prism 9. In vitro data showing biological replicates were presented as mean or median and statistically analyzed using the Mann-Whitney test or Kruskal–Wallis test. In vitro data showing technical replicates were presented as mean ± SD and statistically analyzed using the unpaired *t*-test or ordinary one-way ANOVA test. In vivo data were presented as mean ± SD and statistically analyzed using the ordinary two-way ANOVA test. *P*-values are as follows: not significant (ns) > 0.05, * ≤ 0.05, ** ≤ 0.01, *** ≤ 0.001, and **** ≤ 0.0001.

## Supplementary information

Revised Supplementary data

Revised Original western blot data

Source data for Tumor size

## Data Availability

All data needed to evaluate the conclusions in the paper are present in the article and/or Supplementary Materials. Additional data are available upon request from the corresponding author.
