# Supplementary material for: PARP inhibition-associated heterochromatin confers increased DNA replication stress and vulnerability to ATR inhibition in SMARCA4-deficient cells
Source: Cell Death Discov. 2025 Jan 28;11:31. doi: 10.1038/s41420-025-02306-1 (PMC11775187; doi:10.1038/s41420-025-02306-1)
Supplement: Supplementary file 1 — Revised Supplementary data [file 41420_2025_2306_MOESM1_ESM.pdf]

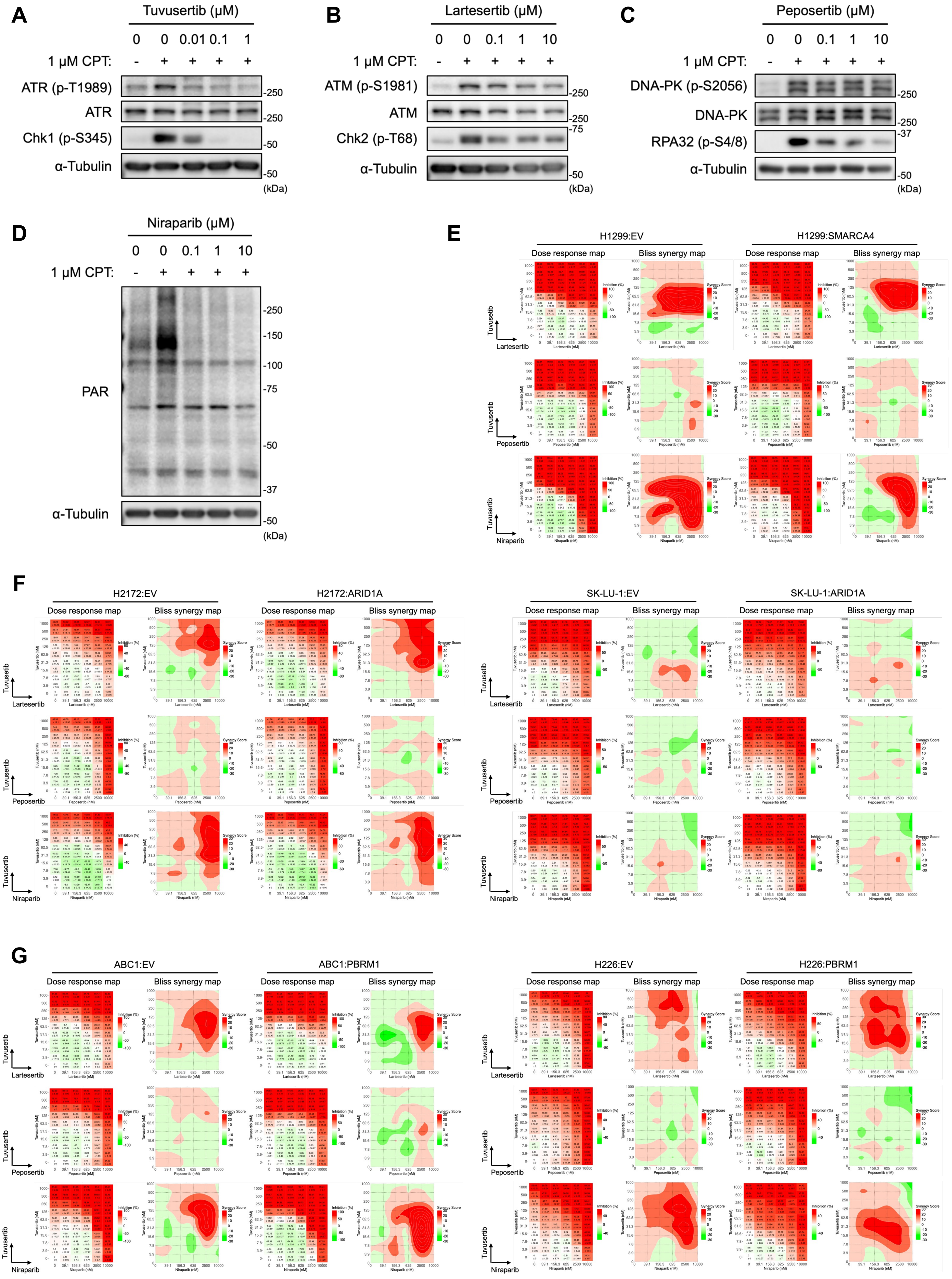

Yano K. et al., Supplementary figure 1

H

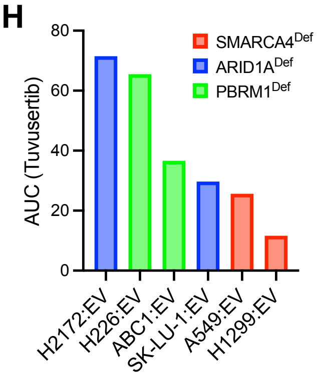

Supplementary figure 1.
(A-D) Western blot analyses of H1975 cells. Cells were treated with 1 μM camptothecin (CPT) and tuvusertib, lartesertib, nidisertib, or niraparib for 2 h, then carried on western blot analysis. α-Tubulin was detected as loading control.
(E) Dose response and Bliss synergy maps of H1299 cells treated with tuvusertib (0, 3.9-1000 nM) in combination with lartesertib (0, 39.1-10000 nM) , peposertib (0, 39.1-10000 nM), or niraparib (0, 39.1-10000 nM). Cells were treated with these combined inhibitors for 4 days, then were carried on cell viability assay. These maps indicate the mean of three independent experiments.
(F) Dose response and Bliss synergy maps of H2172 and SK-LU-1 cells treated with tuvusertib (0, 3.9-1000 nM) in combination with lartesertib (0, 39.1-10000 nM) , peposertib (0, 39.1-10000 nM), or niraparib (0, 39.1-10000 nM). Cells were treated with these combined inhibitors for 4 days, then were carried on cell viability assay. These maps indicate the mean of three independent experiments.
(G) Dose response and Bliss synergy maps of ABC1 and H226 cells treated with tuvusertib (0, 3.9-1000 nM) in combination with lartesertib (0, 39.1-10000 nM) , peposertib (0, 39.1-10000 nM), or niraparib (0, 39.1-10000 nM). Cells were treated with these combined inhibitors for 4 days, then were carried on cell viability assay. These maps indicate the mean of three independent experiments.
(H) Area under the curve (AUC) of the sensitivity to tuvusertib alone in the cell viability screening shown in Fig. 1B.

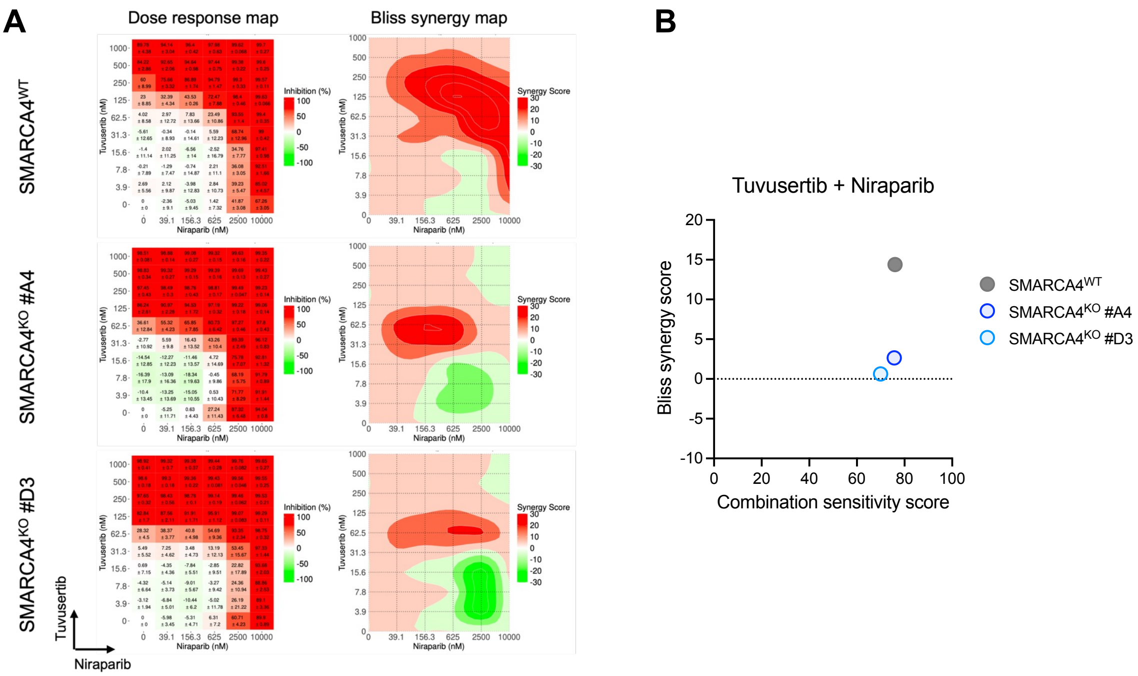

Supplementary figure 2.
(A) Dose response and Bliss synergy maps in the experiment shown in Fig. 2B.
(B) Combination sensitivity score-Bliss synergy score plots of tuvusertib/niraparib combination in the experiment shown in Fig. 2B.

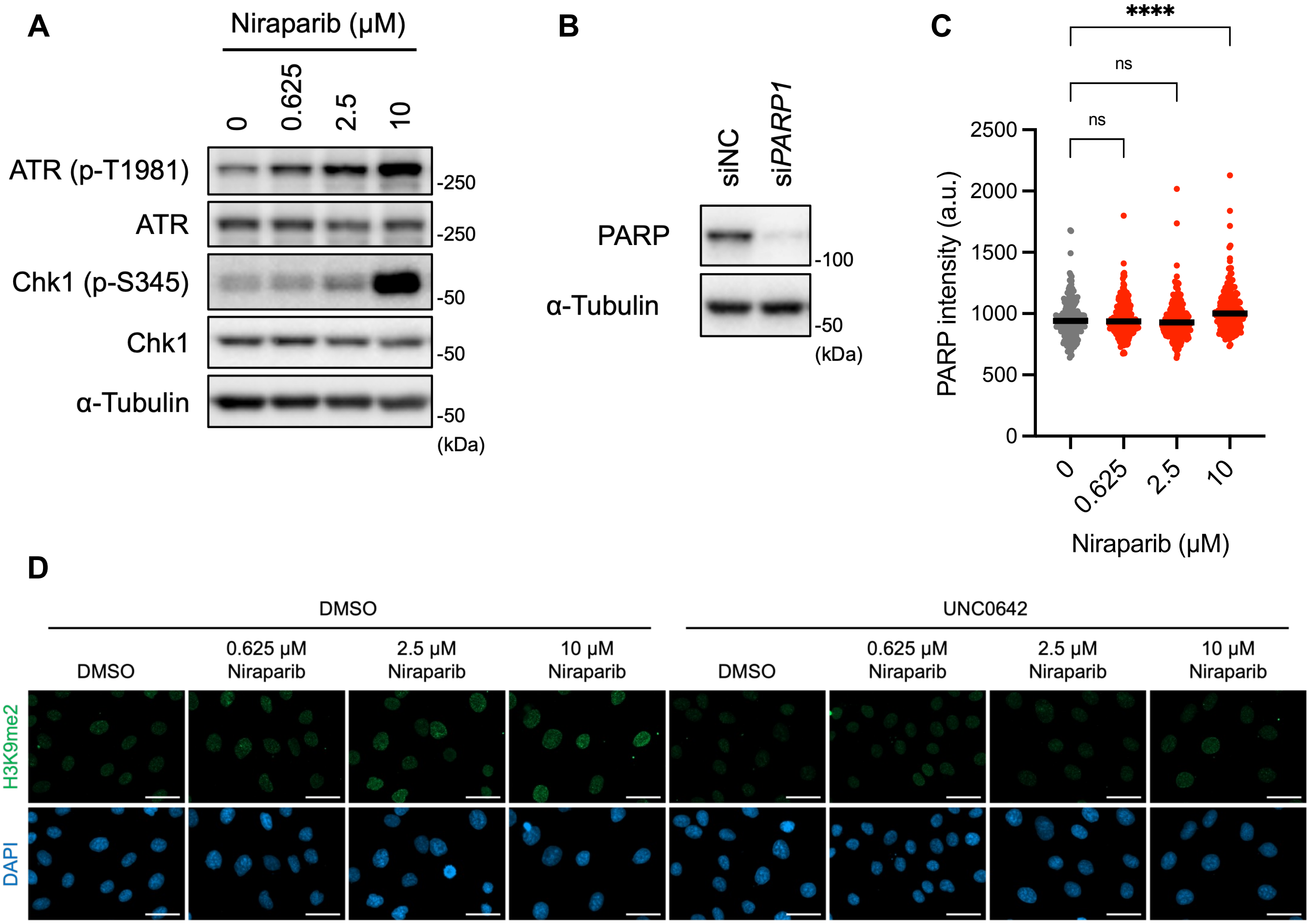

Supplementary figure 3.
(A) Western blot analysis of H1975 cells. Cells were treated with niraparib for 24 h, then carried on western blot analysis. α-Tubulin was detected as loading control.
(B) Western blot analysis of H1975 cells 48 h after transfection with siRNA. α-Tubulin was detected as loading control.
(C) Dot plot of PARP mean intensity per nuclei in H1975 cells. Cells were treated with niraparib for 24 h, then were carried on immunofluorescence analysis. The graph indicates the median of 200 cells and statistical significance by Kruskal-Wallis test with Dunn's multiple comparisons test. P-value: not significant (ns) > 0.05 and **** ≤ 0.0001.
(D) Representative immunofluorescent images of each samples show each and merged images of DAPI (blue), H3K9me2 (green) in the experiment show in Fig. 3F. Cells were treated with 62.5 nM tuvusertib and 625 nM niraparib each alone and combination for 48 h, then carried on immunofluorescence analysis. Scale bars indicate 50 μm.

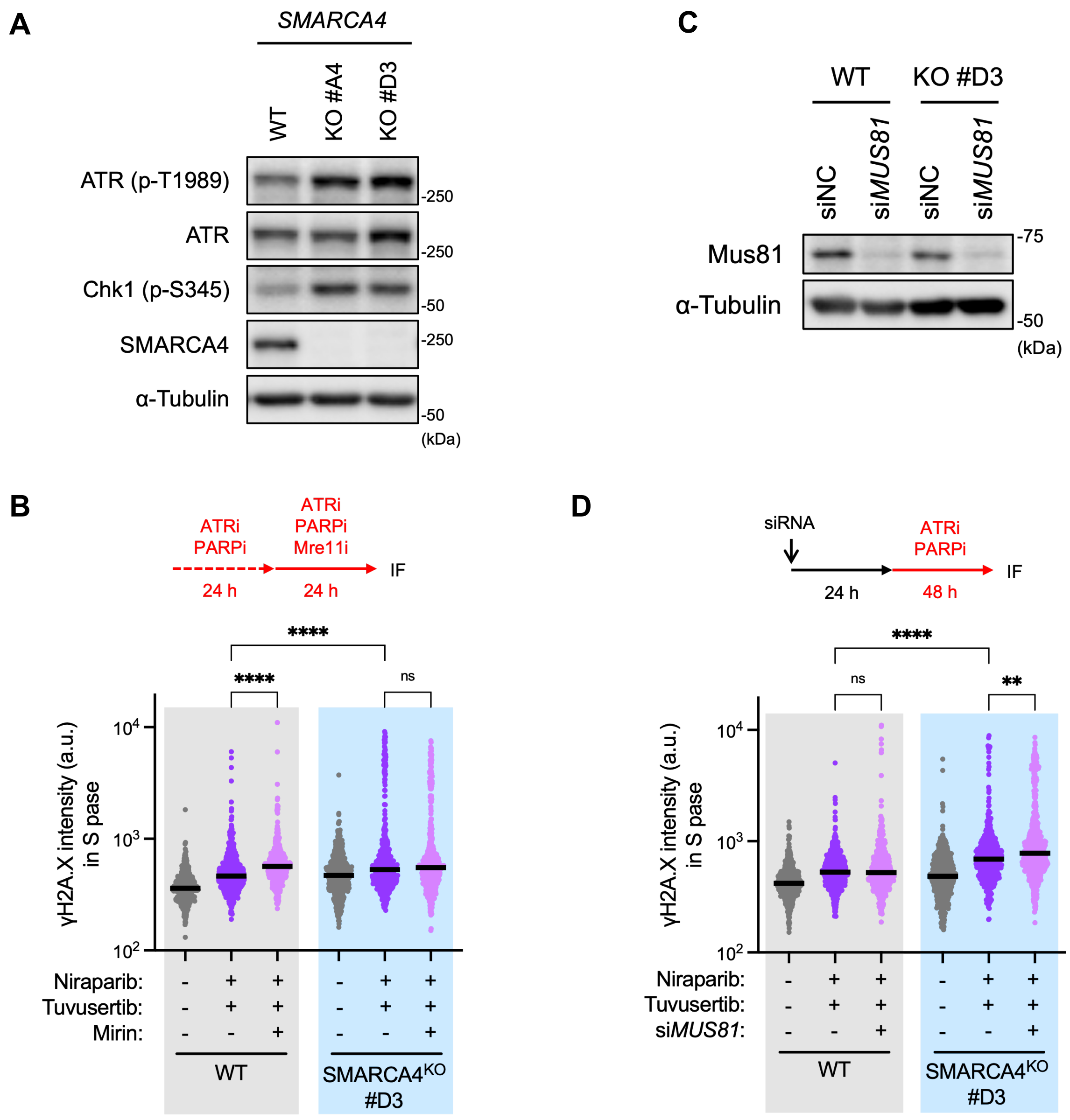

Supplementary figure 4.

(A) Western blot analysis of SMARCA4^{WT} and SMARCA4^{KO} H1975 cells. α-Tubulin was detected as loading control.

(B) Dot plot of γH2A.X mean intensity per nuclei in S phase cells. Cells were treated with 62.5 nM tuvusertib and 625 nM niraparib for 48 h and 25 μM Mirin for 24 h prior to immunofluorescence analysis. PCNA-positive cells were defined as S phase cells. The graph indicates the median of 500 cells and statistical significance by Kruskal-Wallis test with Dunn's multiple comparisons test.

(C) Western blot analysis of SMARCA4^{WT} and SMARCA4^{KO} H1975 cells 72 h after transfection with siRNA. α-Tubulin was detected as loading control.

(D) Dot plot of γH2A.X mean intensity per nuclei in S phase cells. Cells were transfected with siRNA for 24 h, then were treated with 62.5 nM tuvusertib and 625 nM niraparib for 48 h prior to immunofluorescence analysis. PCNA-positive cells were defined as S phase cells. The graph indicates the median of 500 cells and statistical significance by Kruskal-Wallis test with Dunn's multiple comparisons test.

Supplementary Table S1. Gene mutations and protein expression in LUAD cells.

			SMARCA4		ARID1A		PBRM1	
**Cell line**	**Distributors***	**No.**	**Mutation**	**Protein**	**Mutation**	**Protein**	**Mutation**	**Protein**
**A549**	ATCC	CCL-185	p.Q729fs	Absent	N/A	Present	N/A	Present
**H1299**	ATCC	CRL-5803	p.Y560fs	Absent	N/A	Present	N/A	Present
**H2172**	ATCC	CRL-5930	N/A	Present	p.F1720fs	Absent	N/A	Present
**SK-LU-1**	ATCC	HTB-57	N/A	Present	p.G1251fs	Absent	N/A	Present
**ABC1**	JCRB	JCRB0815	N/A	Present	N/A	Present	Large deletion	Absent
**H226**	ATCC	CRL-5826	N/A	Present	N/A	Present	p.Q1559Ter	Absent
**H1975**	ATCC	CRL-5908	N/A	Present	N/A	Present	p.L297I	Present

*ATCC: American Type Culture Collection; JCRB: Japanese Collection of Research Bioresources
