# Supplementary figures and images for: PARP inhibition-associated heterochromatin confers increased DNA replication stress and vulnerability to ATR inhibition in SMARCA4-deficient cells
Source: Cell Death Discov. 2025 Jan 28;11:31. doi: 10.1038/s41420-025-02306-1 (PMC11775187; doi:10.1038/s41420-025-02306-1)

Fig. 1A

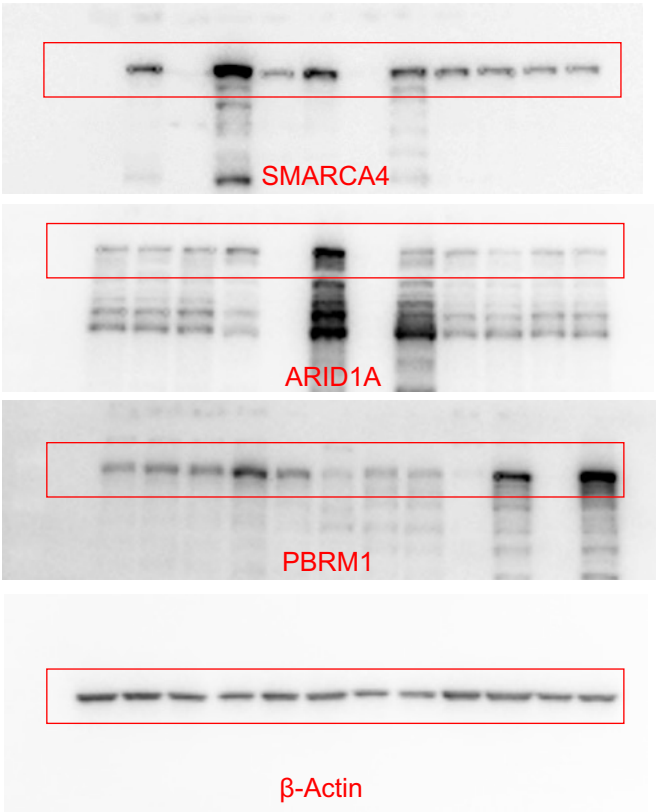

Fig. 2A

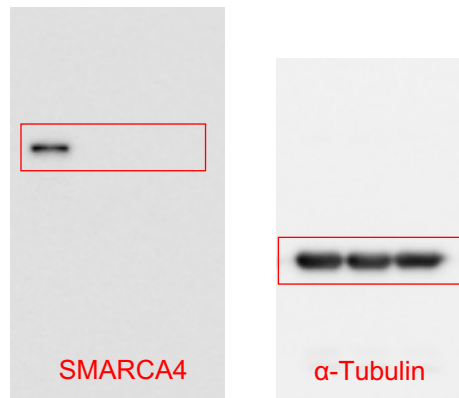

Fig. 4D

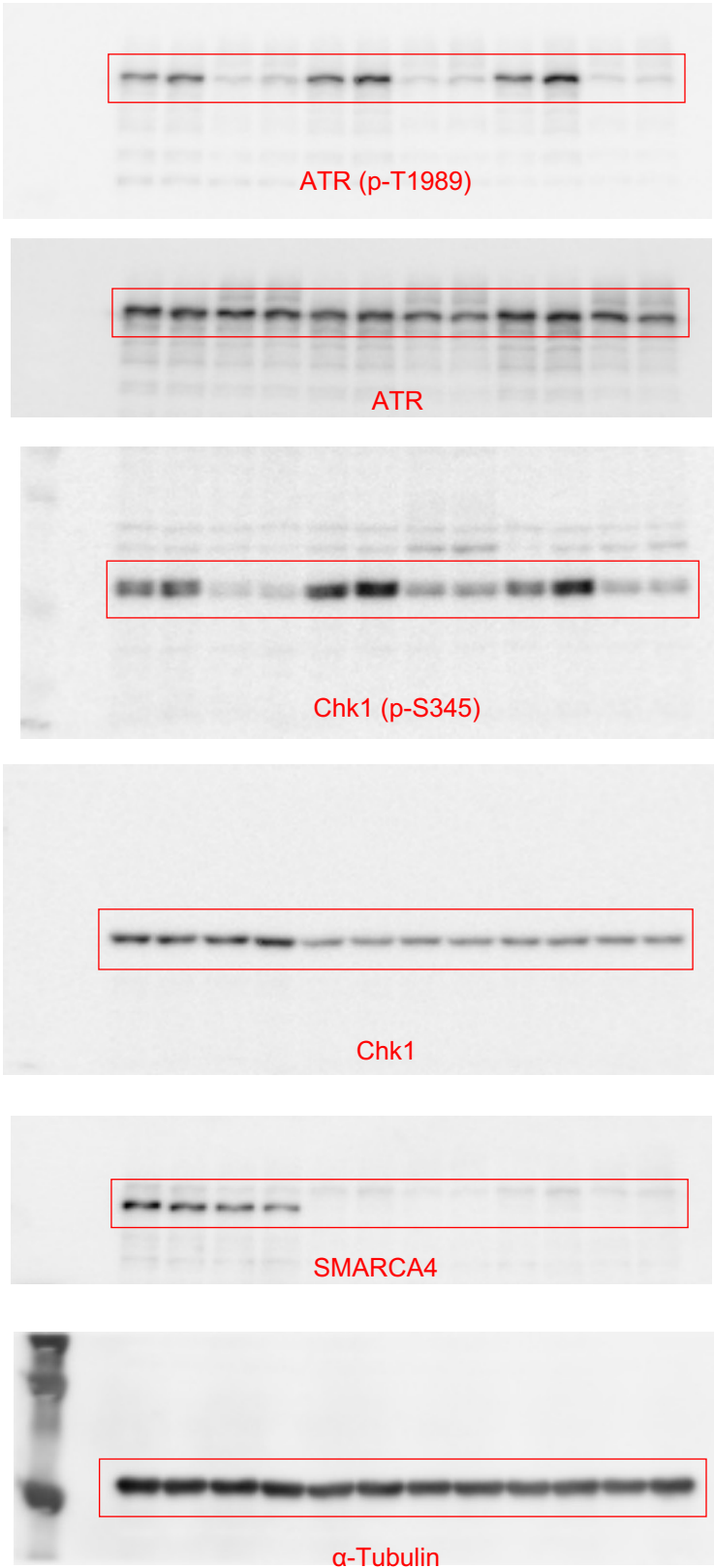

Fig. S1A

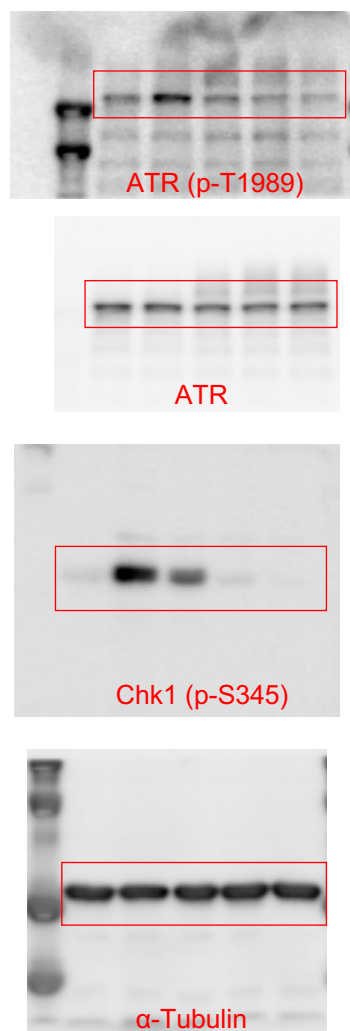

Fig. S1B

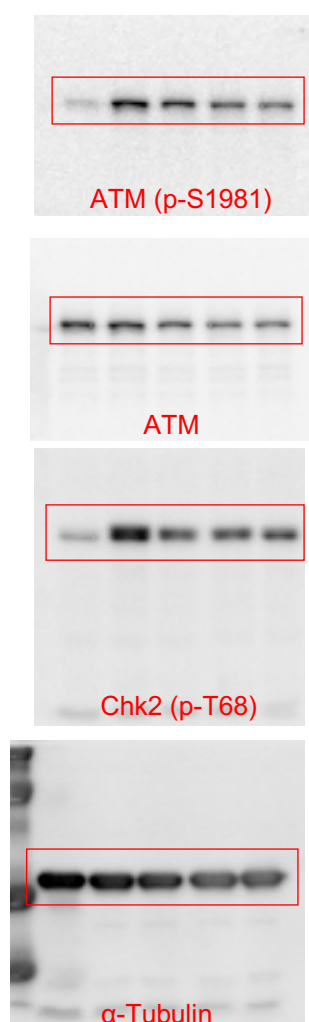

Fig. S1C

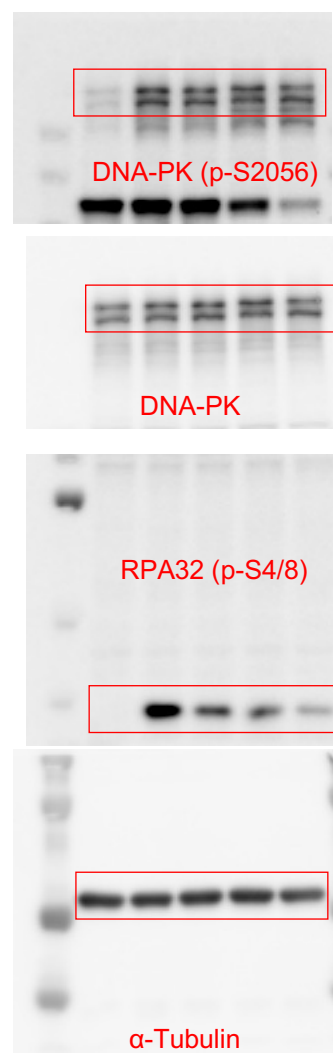

Fig. S1D

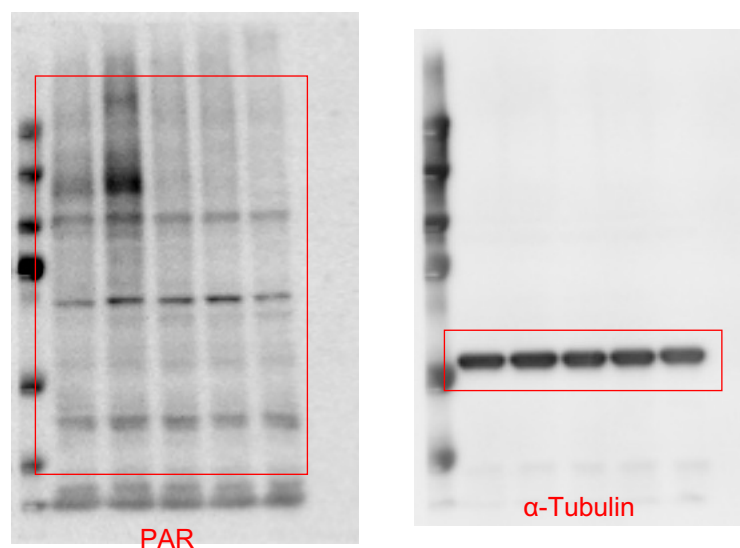

Fig. S3A

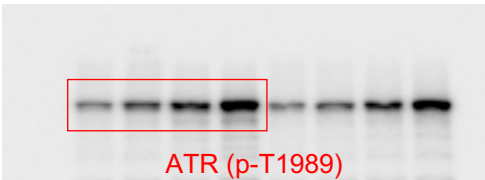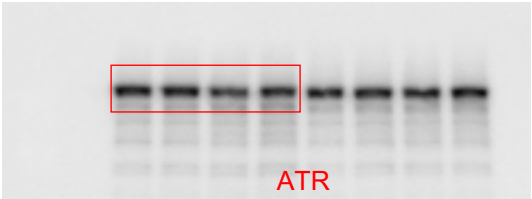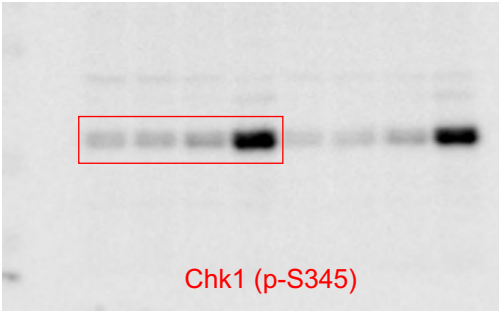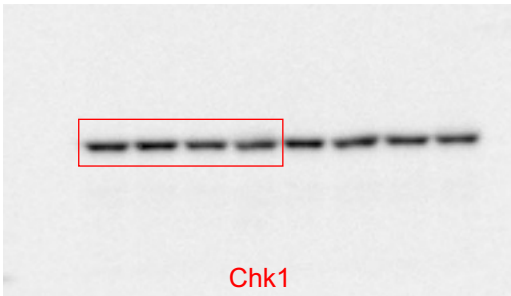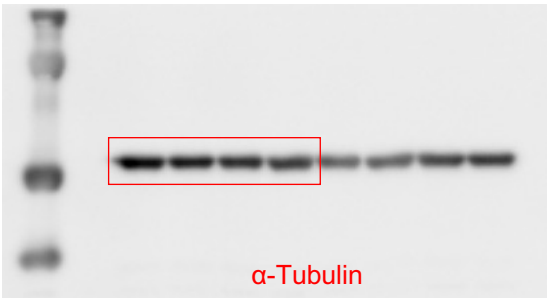

Fig. S3B

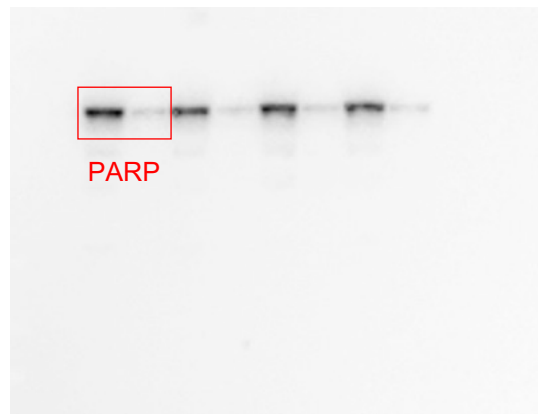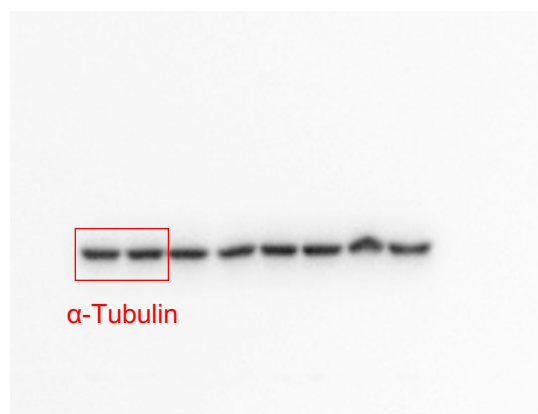

Fig. S4A

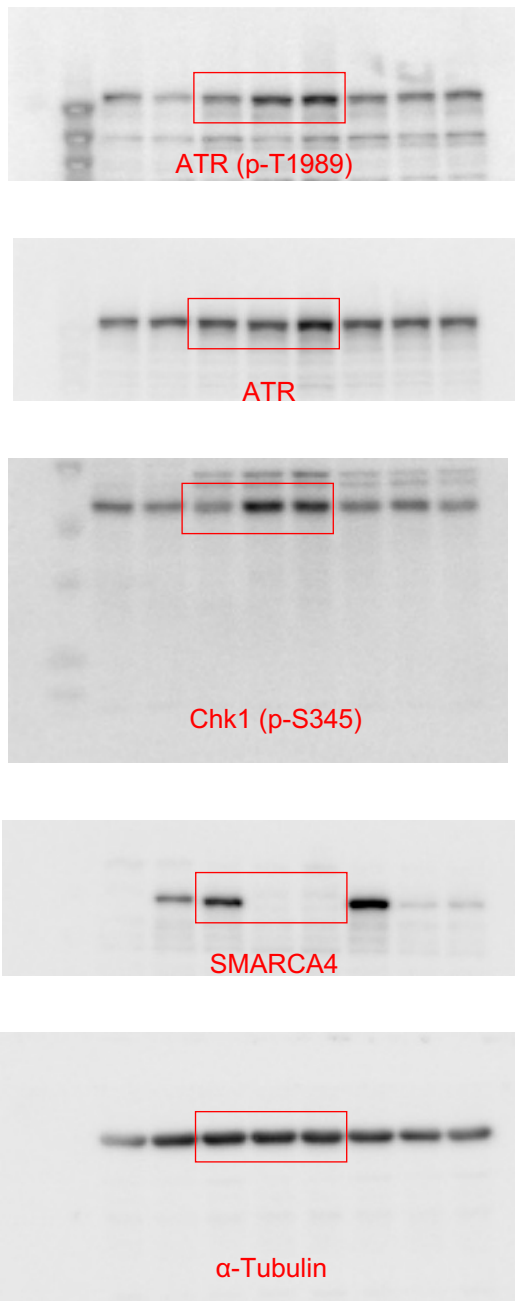

Fig. S4C

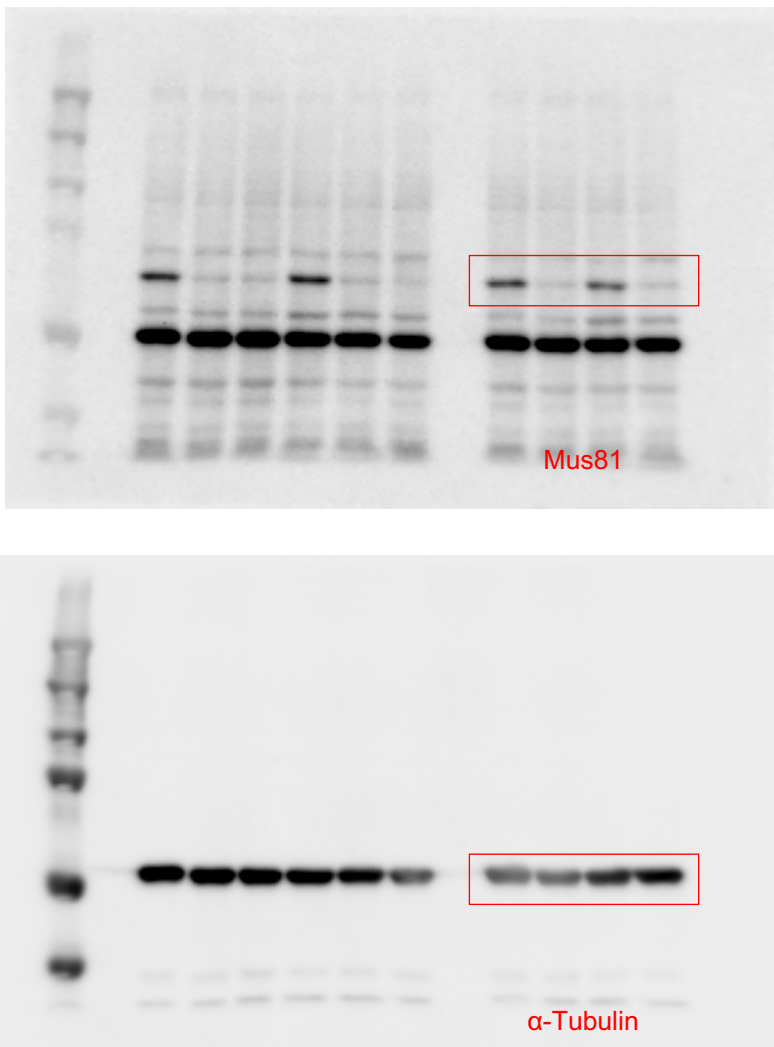

Supplement: Supplementary file 2 — Revised Original western blot data [file 41420_2025_2306_MOESM2_ESM.pdf]
